# Epigenomic Regulators *Elongator Complex Subunit 2* and *Methyltransferase 1* Differentially Condition the Spaceflight Response in Arabidopsis

**DOI:** 10.3389/fpls.2021.691790

**Published:** 2021-09-13

**Authors:** Anna-Lisa Paul, Natasha Haveman, Brandon Califar, Robert J. Ferl

**Affiliations:** ^1^Plant Molecular and Cellular Biology Program, University of Florida, Gainesville, FL, United States; ^2^Horticultural Sciences Department, University of Florida, Gainesville, FL, United States; ^3^Interdisciplinary Center for Biotechnology Research, University of Florida, Gainesville, FL, United States; ^4^Genetics Institute, University of Florida, Gainesville, FL, United States; ^5^Office of Research, University of Florida, Gainesville, FL, United States

**Keywords:** spaceflight adaptation, DNA methylation, epigenetic, space biology, microgravity, elongator complex subunit 2, methyltransferase 1, methylation mutants

## Abstract

**Background:** Plants subjected to the novel environment of spaceflight show transcriptomic changes that resemble aspects of several terrestrial abiotic stress responses. Under investigation here is whether epigenetic modulations, similar to those that occur in terrestrial stress responses, have a functional role in spaceflight physiological adaptation. The Advanced Plant Experiment-04 – Epigenetic Expression experiment examined the role of cytosine methylation in spaceflight adaptation. The experiment was conducted onboard the International Space Station, and evaluated the spaceflight-altered, genome-wide methylation profiles of two methylation-regulating gene mutants [methyltransferase 1 (*met1-7)* and elongator complex subunit 2 (*elp2-5*)] along with a wild-type Col-0 control.

**Results:** The *elp2-5* plants suffered in their physiological adaptation to spaceflight in that their roots failed to extend away from the seed and the overall development of the plants was greatly impaired in space. The *met1-7* plants suffered less, with their morphology affected by spaceflight in a manner similar to that of the Col-0 controls. The differentially expressed genes (DEGs) in spaceflight were dramatically different in the *elp2-5* and *met1-7* plants compared to Col-0, indicating that the disruptions in these mutants resulted in a reprogramming of their spaceflight responses, especially in *elp2-5*. Many of the genes comprising the spaceflight transcriptome of each genotype were differentially methylated in spaceflight. In Col-0 the majority of the DEGs were representative of the now familiar spaceflight response, which includes genes associated with cell wall remodeling, pathogen responses and ROS signaling. However, the spaceflight transcriptomes of *met1-7* and *elp2-5* each presented patterns of DEGs that are almost completely different than Col-0, and to each other. Further, the DEGs of the mutant genotypes suggest a more severe spaceflight stress response in the mutants, particularly in *elp2-5*.

**Conclusion:** Arabidopsis physiological adaptation to spaceflight results in differential DNA methylation in an organ-specific manner. Disruption of Met1 methyltransferase function does not dramatically affect spaceflight growth or morphology, yet *met1-7* reprograms the spaceflight transcriptomic response in a unique manner. Disruption of *elp2-5* results in poor development in spaceflight grown plants, together with a diminished, dramatically reprogrammed transcriptomic response.

## Introduction

Plants cope with environmental changes by reprogramming gene expression and metabolic processes necessary for growth, development, and survival (e.g., Hirayama and Shinozaki, 2010; Sewelam et al., 2014; Lopez-Ruiz et al., 2020). The adaptability of a genotype to changing environmental conditions is therefore determined by its genome and gene activity, both of which are in turn influenced by epigenetic factors such as DNA methylation (e.g., Dowen et al., 2012; Zhang et al., 2018). The APEX-04 EPEX spaceflight experiment investigated the role of specific epigenomic changes in determining the physiological adaptation of plants to the spaceflight environment.

DNA methylation profiles within a genome are dynamic and complex, yet integral to plant growth, development, and stress responses (reviewed in: Bartels et al., 2018). Many terrestrial abiotic stresses, such as salt stress, heat stress, drought, water stress, and phosphate starvation induce epigenetic changes that aid in the adaptation process (e.g., Labra et al., 2002; Boyko and Kovalchuk, 2010; Mirouze and Paszkowski, 2011; Bilichak et al., 2012; Colaneri and Jones, 2013; Tricker et al., 2013; Yong-Villalobos et al., 2015, 2016; Hewezi et al., 2017, 2018; Bartels et al., 2018; Kenchanmane Raju et al., 2018; Beyrne et al., 2019; Ashapkin et al., 2020; Akhter et al., 2021; Korotko et al., 2021; Laanen et al., 2021; Villagomez-Aranda et al., 2021). The genes differentially expressed in response to spaceflight share similarities with many documented terrestrial responses. Hallmarks of spaceflight responses include differential expression of genes involved in pathways associated with cell wall remodeling, reactive oxygen species (ROS), pathogen attacks, wounding, salt stress, drought stress, and hormone signaling (Hoson et al., 2002; Gao et al., 2008; Salmi and Roux, 2008; Blancaflor, 2013; Correll et al., 2013; Paul et al., 2013; Zupanska et al., 2013; Ferl et al., 2014; Inglis et al., 2014; Nakashima et al., 2014; Sugimoto et al., 2014; Kwon et al., 2015; Schüler et al., 2015; Zhang et al., 2015; Ferl and Paul, 2016; Herranz et al., 2019; Vandenbrink et al., 2019; Barker et al., 2020; Califar et al., 2020; Kruse et al., 2020; Angelos et al., 2021; Manian et al., 2021b). Plants further respond to spaceflight with changes in DNA methylation, again similarly to the epigenetic effects that occur during terrestrial stresses. Genome-wide DNA methylation and gene expression alterations occurred in plants grown for part of their life cycle in a satellite experiment (Xu et al., 2018). Arabidopsis grown from seed on orbit in the International Space Station (ISS) showed changes in specific DNA methylation contexts, with some of those changes associated with differentially expressed genes (DEGs; Zhou et al., 2019).

The patterns of spaceflight-associated DNA methylation are organ-specific. In comparison to roots, spaceflight leaves show higher methylation levels within the protein-coding genes compared to ground controls (GCs; Zhou et al., 2019). A large proportion of the genes that are differentially expressed and differentially methylated are associated with ROS signaling (Zhou et al., 2019). ROS can act as signaling molecules in responses to an array of plant stressors and there is growing evidence of an interplay between ROS metabolism and epigenetic regulation during acclimation in terrestrial environments (Huang et al., 2019). DNA methylation and other epigenetic modifications have been reported to play a role in regulating the innate immune response and pathogen response (Dowen et al., 2012; Wang et al., 2013; Yu et al., 2013; Tameshige et al., 2015; Jarosz et al., 2020). Many components of the gene networks associated with these pathogen-associated pathways are also differentially expressed by plants in spaceflight (Correll et al., 2013; Paul et al., 2013, 2017; Sugimoto et al., 2014; Kwon et al., 2015; Schüler et al., 2015; Herranz et al., 2019; Barker et al., 2020; Califar et al., 2020; Manian et al., 2021a). This commonality of terrestrial environmental responses with spaceflight responses begged the question: do plants use similar tools to regulate genes in response to spaceflight?

DNA methylation in plants occurs in three main contexts, CG, CHG, and CHH (where H = A, C, or T). Methylation in each context is directly maintained by a distinct pathway and set of enzymes which include: Methyltransferase 1 (MET1), decreased DNA methylation 1 (DDM1)1, and variant in methylation in the CG context, SUVH4-deposited H3K9me2 and CHROMOMETHYLASE 3 (CMT3) in the CHG context, and CMT2 in the CHH context (Chevalier et al., 2005; Hsieh et al., 2012; Pikaard, 2013). In addition to these direct enzymatic regulators of DNA methylation, a number of genes indirectly affect DNA methylation through RNA intermediates. RNA-directed DNA methylation facilitates the recruitment of DNA methyltransferases. In plants, small interfering RNAs (siRNAs) direct *de novo* DNA methylation and maintenance of DNA methylation at asymmetrical CHH sites through the polymerase II (Pol II)-related RNA polymerases Pol IV and Pol V (Matzke et al., 2015). In Arabidopsis, the siRNA effector ARGONAUTE4 (AGO4) exists in a complex with domains rearranged methyltransferase (DRM) for methylation of the template strand for RNA polymerase V-mediated non-coding RNA transcripts (Zhong et al., 2014). The Elongator complex is a co-factor of RNA Pol II, and ELP2 is the most likely subunit to interact with the siRNA machinery (Woloszynska et al., 2016). To explore the effects of these two DNA methylation pathways in conditioning the spaceflight response, we chose MET1 as a representative of the proteins directly involved in methylation and ELP2 as a model regulator that affects methylation during stress responses through RNA intermediates and regulation of methyltransferases.

The cytosine DNA methyl transferase gene, Met1, is directly involved in the maintenance of cytosine methylation in Arabidopsis, particularly at CG sites (Kankel et al., 2003; Rigal et al., 2016). MET1 is one of several methylation enzymes that also modify the epigenome of plants as part of stress responses (Naydenov et al., 2015; Yong-Villalobos et al., 2015; Dhami and Cazzonelli, 2020). The role MET1 plays in maintaining the CG methylation profile is also important for the subsequent inheritance of those epigenomic changes (Saze et al., 2003). Loss of MET1 function non-specifically enhances resistance to bacterial infections (Kankel et al., 2003; Dowen et al., 2012). Many of the genes differentially regulated in methylation-mutant *met1-3* lines (Supplementary Material, Dowen et al., 2012) show a substantial overlap with genes that are differentially expressed in response to spaceflight (Paul et al., 2013, 2017; Zhou et al., 2019) including genes involved in defense, transcription, response to hormone stimulus and phosphorylation. These methylation mutant transcriptome profiles establish that differential methylation in the genome is central to facilitating stress responses in Arabidopsis (Dowen et al., 2012; Zhang, 2012), suggesting the hypothesis that the stress response elicited by spaceflight in Arabidopsis may also have an epigenetic component that could involve regulation by MET1.

The Elongator complex is composed of six protein subunits that are highly conserved among eukaryotes; it acts as a co-factor of RNA Pol II, and has several unique cellular functions, including tRNA modification, DNA modification, and histone acetyltransferase (HAT) activity (Nelissen et al., 2005; Glatt and Muller, 2013; Ding and Mou, 2015; Kolaj-Robin and Seraphin, 2017). Although loss of any component can compromise Elongator complex function (Ding and Mou, 2015) the subunits have independent activity and can also function as sub-complexes (e.g., Glatt and Muller, 2013; Jarosz et al., 2020). Elongator proteins were initially identified in plants as important to various aspects of growth, development, and immune response (Woloszynska et al., 2016). Elongator Proteins ELP3 and ELP2 both contribute epigenomic regulation of gene expression in response to developmental and immune response pathways in Arabidopsis. ELP3 exhibits HAT activity while ELP2 interacts with components of the siRNA machinery (Woloszynska et al., 2016). In plants, siRNAs can induce cytosine methylation by recruitment of the DNA methyl transferase DRM2 through RNA-directed DNA methylation (e.g., Xie and Yu, 2015), and it is by this mode of action that ELP2 regulates plant development and stress responses (reviewed in: Jarosz et al., 2020).

ELP2 is associated with terrestrial pathogen responses (e.g., Wang et al., 2013) and plays a role in root development (e.g., Jia et al., 2015), The role of ELP2 in plant pathogen defense is relevant to the spaceflight response in plants as many of the genes comprising the spaceflight transcriptome are also commonly associated with terrestrial pathogen responses (e.g., Paul et al., 2013, 2017; Zhou et al., 2019; Barker et al., 2020). Functionally, ELP2 is a regulator of NONEXPRESSOR OF PATHOGENESIS-RELATED GENES 1 (NPR1) and accelerates the immune responses in Arabidopsis through epigenetic modification of DNA methylation (DeFraia et al., 2010; Wang et al., 2013; Silva et al., 2017). Deletion of the gene encoding ELP2 has substantial impact on pathogen-induced transcription (DeFraia et al., 2010; Wang et al., 2013; Silva et al., 2017), and also impacts fundamental processes such as root development (Jia et al., 2015) and auxin signaling (Nelissen et al., 2010). Many of the genes differentially expressed between *elp2* mutants and wild-type plants include hallmarks of abiotic stress responses (Zhou et al., 2009). Further, many of the genes differentially expressed in *elp2* mutants are also represented in Arabidopsis spaceflight transcriptomes and spaceflight methylome (e.g., Paul et al., 2013, 2017; Zhou et al., 2019). These roles for ELP2 in pathogen responses and abiotic stresses suggest that ELP2 may also play a role in the epigenetic regulation of the spaceflight response pathways in plants.

The Advanced Plant Experiment 04 – Epigenetic Expression (APEX-04-EPEX) spaceflight experiment reported herein investigated the contribution of epigenomic changes in the physiological adaptation of plants to the spaceflight environment, specifically the role of cytosine DNA methylation in the spaceflight response of Arabidopsis. The experiment was conducted onboard the ISS to evaluate the spaceflight responses of the methylation mutants *met1-7* and *elp2-5* compared to the Col-0 wild-type background of both mutant lines. Growth patterns, genome-wide methylation profiles, and differential gene expression profiles were used to develop an integrated assessment of the interplay of DNA methylation and transcriptional regulation in the response to spaceflight.

## Materials and Methods

### Plant Material and Experimental Design

Three lines of *Arabidopsis thaliana* (Arabidopsis) were used: Columbia-0 (Col-0) wild-type (TAIR CS70000), and Col-0 mutants *met1-7* and *elp2-5* genotype. Mutants *met1-7* and *elp2-5* previously described in Kanno et al. (2008), Guo et al. (2013), Wang et al. (2013), and were kindly provided by Dr. Zhonglin Mou (Wang et al., 2013). Sterilized seeds from each line were sown aseptically onto Petri dishes (100 mm × 15 mm; Fisher Scientific, Pittsburgh, PA, United States), containing 50 mL of a 0.5% Phytagel-based growth medium supplemented with: 0.5 × Murashige–Skoog salts, 0.5% (w/v) sucrose, and 1 × Gamborg’s Vitamin Mixture, and then sealed with breathable tape (3M Micropore^®^, Maplewood, MN, United States; e.g., Califar et al., 2020). Seeded plates were prepared to maintain seed dormancy with a combination of far-red light treatment and light-tight wrapping in Duvetyne^TM^ cloth until insertion into Veggie growth facility on orbit. Dormancy preparation details described in Sng et al. (2014), Fitzgerald et al. (2016), Califar et al. (2020).

Ten plates (nine for installation in Veggie and one spare) per genotype for each environmental condition (flight and GC) were prepared for launch. The plates were stored at 4°C and remained dormant until their installation into the Vegetable Production System (Veggie) hardware on the ISS, and a comparable set was installed into the Veggie hardware in the ISS environmental simulator (ISSES) chamber at Kennedy space center. Plants in the Veggie hardware (both on the ISS and in the ISSES chamber) were exposed to constant light conditions of 100–135 μmoles/m^2^s PAR for 11 days before being harvested into Kennedy space center fixation tubes (KFTs) and fixed in RNAlater^TM^ (Ambion, Grand Island, NY, United States). All of the plants from each plate were harvested into individual KFTs. The KFTs were then stowed at −80°C in the MELFI freezer aboard the ISS. The comparable GC samples were also harvested into KFTs and stored in a standard −80°C freezer. All samples were kept frozen until delivery to the laboratory for analysis. Each plate was intended as a biological replicate and kept separate in tissue preparation operations, but the mass values drove the decision to allow a combination of two plates to comprise a replicate, which resulted in a total of four biological replicates for each of the genotypes and treatments for the analyses. Leaf and root tissues from each sample were dissected using an Olympus stereo-microscope. FromBioanalyzer and quantified with the each plate, the materials from three individual plants were pooled for RNA extraction and transcriptome analyses, and materials from 10 to 15 individual plants from that same plate were allocated for DNA extraction. Molecular analyses were performed on leaf and root tissues dissected from each of the three genotypes. One plate was lost, (*met1-7*, spaceflight C6) and so one of the spaceflight *met1-7* replicates was composed of plants from a single plate rather than two plates. Whole-genome bisulfite sequencing (WGBS) was performed for the methylome analysis and RNA sequencing (RNASeq) for the transcriptome analysis. To summarize, for each genotype under each environmental condition, a total seven (spaceflight *met1-7*) or eight (all other genotypes and treatments) plates were used to conduct WGBS and RNASeq analyses, four biological replicates for each assay.

### Experiment Operations for Spaceflight and Ground Controls

**FIGURE 1:**
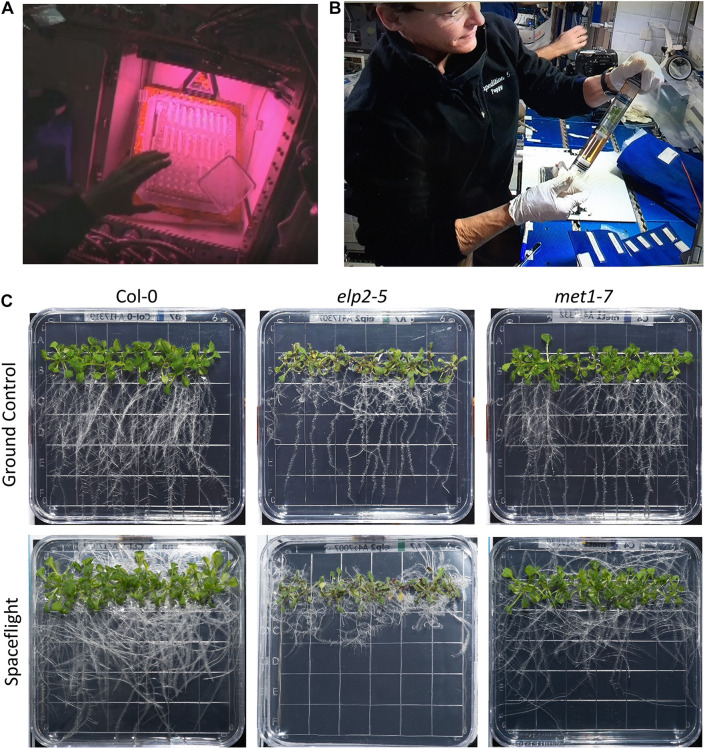
On orbit operations and comparison of plant growth in both environmental conditions. **(A)** Astronaut Peggy Whitson inserts seeded plates into the Veggie growth hardware on the ISS. The pinkish lighting is derived from the mixture of red and blue LED growth lights (also Supplementary File 1). **(B)** Peggy Whitson harvests the 11 day old seedlings to RNAlater-filled KFTs at the MWA on the ISS. **(C)** Representative examples of each of the three genotypes (Col-0 wild-type, *elp2*, and *met1-7*) grown for 11 days in the Veggie hardware in the ISS Environment simulation chamber (ISSES) for the ground controls (top row) and grown for 11 days in Veggie on the ISS for the spaceflight environment (bottom row; also Supplementary File 3).

The plates comprising the NASA APEX-04-EPEX experiment was launched on the SpaceX mission CRS-10 to the ISS. NASA astronauts Peggy Whitson and Shane Kimbrough (expedition 49/50) managed the experiment from insertion into the Veggie growth hardware to harvest (Figures 1A,B). Images and videos of ISS operations are collected in Supplementary Files 1, 2.

The workflow of the orbital operations was recapitulated for the GCs in the ISSES chamber with a 24 h delay. The delay enabled a precise ground replication of minor changes in daily ISS environmental parameters and crew operations scheduling. Crew operations included the de-stowing of the plates and insertion into Veggie (Figure 1A), daily high-resolution photography of representative plates were taken with a hand-held DSLR camera, and the harvest and fixation of the plates on the final day of growth. Photography and harvests were conducted on the maintenance work area. All plates were photographed before harvest, and representatives of each genotype presented in Figure 1C. The complete set of spaceflight and GC photographs are supplied in the Supplementary File 3. The images were used to assess the growth morphology and general health of the plants.

### Genomic DNA Isolation and Whole-Genome Bisulfite Sequencing Library Preparation

DNA extraction was done using a modified phenol/chloroform protocol (LeFrois et al., 2016) and genome-wide bisulfite sequencing was performed using a similar procedure as that described by Wang et al. (2013), Zhou et al. (2019). Briefly, 700–1,700 ng of genomic DNA (>5 Kb in length) observed on the TapeStation Genomic Screen Tape (Agilent) was processed for sequencing library construction. DNA was transferred into 6 × 16 mm glass microtubes with AFA fiber and pre-slit snap caps (Cat# 520045, Covaris, Inc.) and sheared into an average fragment size of ∼400 bp using the Covaris S220 ultrasonic disruptor. Short DNA fragments (<100 bp) were removed using AMPure magnetic beads (Cat# A63881, Beckman Coulter) at a 1:1 bead to sample ratio. 100–250 ng of clean, fragmented DNA was used for the Illumina sequencing library construction. Both the NEBNext^®^ Ultra^TM^ II DNA Illumina construction kit (Cat# E7645S, NEB) and the Illumina-specific methylated and dual-index barcoded adaptors (Cat# E7600S NEB) were used as described in the manufacturer’s guidelines. Illumina libraries (containing methylated adaptors) were subjected to sodium bisulfite treatment using the EZ DNA Methylation Direct kit (ZYMO Research, Cat #D5020) according to the manufacturer’s instructions. The resulting libraries were enriched by a 13–15 cycle amplification using a uracil-insensitive polymerase (EpiMark hot start Taq polymerase, NEB, Cat #M0490S). The amplified library products were separated on a 2% agarose gel from which library fragments in the 250–500 bp range were excised (QIAquick gel extraction kit, Cat# 28704, QIAGEN) and AMPure purified (Cat# A63881, Beckman Coulter). Gel staining was done with SYBR Safe (Life Technologies) and visualized on a blue light transilluminator (Life Technologies) to avoid UV damage to the DNA. The final libraries were quantified by the RNA concentration was determined on Qubit^®^ 2.0 Fluorometer (ThermoFisher/Invitrogen, Grand Island, NY, United States), sized on the Agilent TapeStation (DNA5000 Screen Tape) and by qPCR with the Kapa SYBR Fast qPCR reagents (Cat# KK4824, Kapa Biosystems) with monitoring on an ABI7900HT real-time PCR system (LifeTechnologies). The average library size was 350 bp. Care was taken to generate WGBS libraries that were approximately the same size as the RNASeq libraries. Sequencing was performed at the ICBR NextGen Sequencing Core^1^.

### Total RNA Isolation and RNASeq Library Construction

RNA extraction was performed using RNeasy Plant Mini Kit (Qiagen, Hilden, Germany) according to the manufacturer’s guidelines. RNA concentration was determined on a Qubit^®^ 2.0 Fluorometer, RNA quality was assessed using the Agilent 2100 Bioanalyzer (Agilent Technologies, Inc.). The RIN numbers of the total RNA used for RNASeq library construction are between 7.1 and 9.3. Basically, 2 μL of 1:200 diluted RNA spike-in ERCC (half amount of suggested in the ERCC user guide: Cat# 4456740) spike to 1,000 ng of total RNA followed by mRNA isolated using the NEBNext Poly(A) mRNA Magnetic Isolation Module (New England Biolabs, catalog # E7490). This was followed by RNA library construction with the NEBNext Ultra Directional RNA Library Prep Kit (New England Biolabs, catalog #E7420) according to the manufacturer’s user guide. Briefly, RNA was fragmented in NEBNext First Strand Synthesis Buffer via incubation at 94°C for the desired time. This step was followed by first-strand cDNA synthesis using reverse transcriptase and Oligo(dT) primers. Synthesis of ds-cDNA was performed using the second strand master mix provided in the kit, followed by end-repair and adaptor ligation. At this point, Illumina adaptors were ligated to the sample. Finally, each library (uniquely barcoded) was enriched by 10 cycles of amplification, and purified with Agencourt AMPure beads (Beckman Coulter, catalog # A63881). 48 barcoded libraries were sized on the Bioanalyzer and quantified with the Qubit^®^ 2.0 Fluorometer. Finally, these 48 individual libraries were pooled in equimolar concentration. RNASeq libraries were constructed at the UF ICBR Gene Expression Core^2^.

### HiSeq3000 Procedure, Pooled RNASeq, and MethylSeq

Uniquely barcoded libraries were normalized to 2.5 nM and pooled (equimolarly) for sequencing on the HiSeq3000 Illumina sequencer. Bisulfite-converted sequencing libraries were sequenced together with RNASeq libraries (uniquely barcoded) to maximize data output. The RNASeq libraries in the pool served to compensate for the low base diversity of bisulfite-converted genomic libraries. The final library was created by mixing RNASeq vs bisulfite-converted libraries at a 60:40% ratio, with a mere 1% PhiX spike-in. Library pools were processed according to the Illumina protocol (HiSeq3000) for clustering on the cBOT machine. After denaturation, neutralization, and mixing with the ExAmp reagent, the final pool concentration for clustering was 0.25 nM. Sequencing was done using a 2 × 101 cycles format (paired-end configuration). The 48-sample project was sequenced on 12 lanes for a robust reads/lane output.

### MethylSeq Bioinformatics

The short reads from the uniquely barcoded bisulfite-converted genomic libraries were trimmed using Trimmomatic v 0.36 and quality control on the original and trimmed reads was performed using FastQC v 0.11.4 and MultiQC v 1.1 (Andrews, 2010; Bolger et al., 2014; Ewels et al., 2016). The bisulfite-converted reads were aligned to the TAIR10 genome using BSMAP (Xi and Li, 2009). Methylation calling was performed with CSCALL and the differential methylation analysis was performed using the MCOMP program, which is part of the MOABS package (Abe et al., 1997; Sun et al., 2014). Cytosine sites with at least a 10x read coverage in at least two out of the four replicates were included in downstream analyses. Methylated cytosine sites for which the *p*-value of the difference between test and control methylation rates was below 0.01 were considered differentially methylated cytosines (DmCs). In addition, DmCs with a methylation difference > 0 were classified as hypermethylated whereas those < 0 were classified as hypomethylated. DmCs were also categorized based on the characteristics of their genomic locations including the gene body (from transcriptional start site to transcriptional termination site), promoter (2 kb upstream of transcriptional start site), and downstream (2 kb downstream of transcriptional termination site). Differentially methylated regions (DMRs) were defined following the method described in Stroud et al. (2013). Briefly, DMRs were determined by comparing the average methylation levels within a 100 bp window between spaceflight and GCs, and those with statistical significance (*p* < 0.01) were used in the analysis. The reads mapped to the chloroplast reference genome were used to calculate the bisulfite conversion efficiency as previously described (Zhou et al., 2019).

### RNASeq Bioinformatics

The overall quality of the RNASeq sequence data was first evaluated using FastQC (Andrews, 2010, 2018). Low-quality bases were trimmed from the reads using Trimmomatic (Bolger et al., 2014). STAR Aligner was used to map high-quality paired-end reads to TAIR10 genome (Vandenbrink et al., 2016). Gene expression values were calculated from these alignments using RSEM (Li and Dewey, 2011). The expected read counts and fragments per kilobase of transcript per million mapped reads (FPKM) were extracted for further analysis. A generalized linear regression model was built to perform the differential gene analysis using edgeR (Robinson et al., 2010). Prior to the differential expression analysis, hierarchical clustering and principal component analysis (PCA) were conducted to identify potential outliers in the samples. The thresholds for calling significantly DEG were set at, FDR of 0.05, a fold change of greater than 2, and the average FPKM for at least one replicate of each comparison group being higher than 0. DEG lists were analyzed for overlaps using BioVenn (Hulsen et al., 2008). Processing and Analysis of the RNASeq data was performed at the UF ICBR Bioinformatics Core^3^.

### Functional Categories Enrichment

*Arabidopsis thaliana* gene IDs from each list of differentially methylated, differentially expressed genes (DmC-DEGs) output from combined methylomic and transcriptomic data were submitted to g:Profiler using the standard parameters (Raudvere et al., 2019). Lists of gene ontology (GO) terms enriched within each group of DmC-DEGs were trimmed using REVIGO (Supek et al., 2011).

### Statistical Analyses

Original Student’s *t*-tests were done with Bonferroni corrections. Two-Factor ANOVA analyses with replication were performed to demonstrate statistical differences in methylation levels between genotypes and plant organ samples.

## Results

### Col-0 and *met1-7* Exhibited a Typical Spaceflight Growth Morphology, While *elp2-5* Displayed an Unusual Spaceflight Morphology

The three genotypes, Col-0, *met1-7*, and *elp2-5* each demonstrated different growth habits in response to spaceflight. Figure 1C shows a representative plate of each genotype grown on the ISS (spaceflight, FT) and comparable GC. Photos of all the harvested plates are presented in Supplementary File 3.

Col-0 and *met1-7* exhibited a typical growth morphology of Arabidopsis grown in Veggie during spaceflight (Figure 1A). The strong directional growth light gradient in Veggie produces a negative phototropism in roots that results in root growth that largely mimics terrestrial gravitropism (Zhou et al., 2019; Califar et al., 2020). For both Col-0 and *met1-7* the spaceflight (FT) roots adopt a slightly randomized growth habit compared to the GCs, yet extend well away from the site of germination and the stem, and are generally negatively phototropic in response to the “vertical” orientation of the plates relative to the light source in the Veggie growth habitat. The Col-0 and *met1-7* plants presented healthy visual phenotypes, where their leaves were fully expanded and green.

In contrast, *elp2-5* plants exhibit spaceflight growth patterns that were distinct from both Col-0 wild-type and *met1-7*, and did not appear to be responding to the environmental tropic cues directing roots away from the lights in Veggie (Figure 1C). The roots of *elp2-5* in spaceflight did not exhibit any directional growth. Instead, all root growth appeared to occur in random patterns that resulted in most of the root mass staying within 1 cm of the germinated seed. The spaceflight leaves of *elp2-5* tended to be darker, with more of the plants exhibiting reddish coloration typical of anthocyanin production. Several of the *elp2-5* leaves on each of the spaceflight plates were chlorotic.

In spite of any morphological differences among the genotypes, all produced the same biomass in spaceflight as on the ground. No statistically supported differences were observed between flight and GCs in either roots or shoots, for all three genotypes (Supplementary Figure 1).

### Spaceflight Increased Genome-Wide DNA Methylation Levels in *met1-7* and *elp2-5* but Not in the Wild-Type Col-0 Plants

**FIGURE 2:**
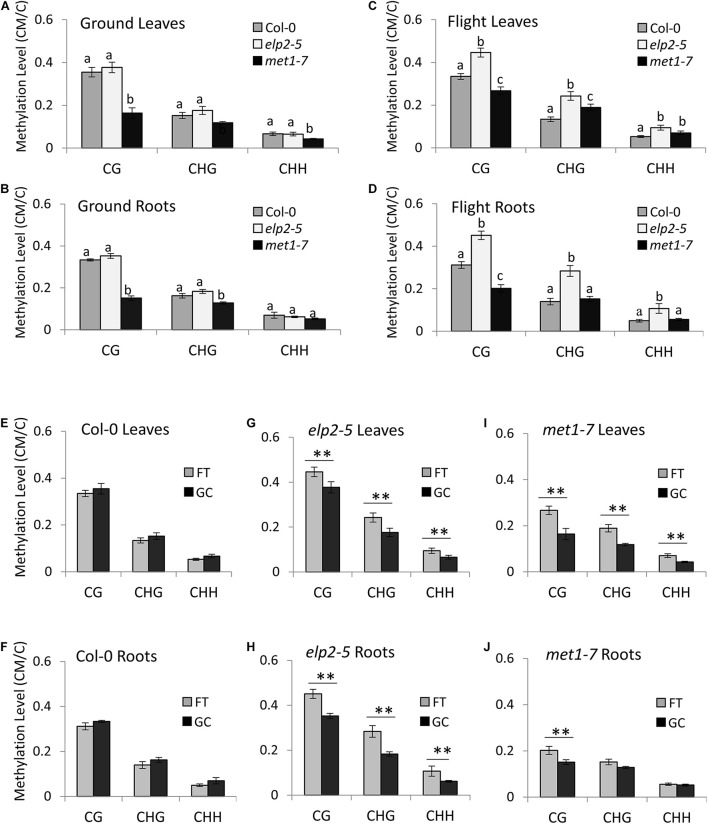
Average genome-wide methylation level profiles in spaceflight and on the ground. **(A–D)** A comparison of the average genome-wide methylation levels (CG, CHG, and CHH) across the three genotypes is illustrated in leaves grown on the ground **(A)**, roots grown on the ground **(B)**, leaves grown in spaceflight **(C)**, and roots grown in spaceflight **(D)**. Statistical analyses were performed using two-sample *t-*test with Bonferroni corrections. Bar graphs with different letters show significant differences (*p* < 0.01). In addition, Two-Factor ANOVA analyses with replication showed that the relationships between methylation contexts were dependent on the samples (*p* < 1.0E-10). **(E–J)** Average genome-wide methylation levels are shown of each genotype (Col-0 wild-type, *elp2*, and *met1-7*) and tissue type (leaves or roots) in spaceflight (FT) and ground controls (GC). Data represent the mean of four independent samples and ^∗∗^ indicate significance (*p* < 0.01, two-sample *t-*test with Bonferroni corrections) difference between flight and ground controls in each of the methylation contexts (CG, CHG, and CHH).

In the GC plant leaves, the methylation level of *met1-7* was statistically lower than that of Col-0 and *elp2-5* plants in all three methylation contexts. Col-0 and *elp2-5* and plants showed similar genome-wide methylation levels (Figures 2A,B). In the GC plant roots, the methylation level of *met1-7* was statistically lower than that of Col-0 and *elp2-5* plants in the CG and CHG contexts. In the CHH context, there were no statistical differences in methylation among genotypes.

In spaceflight plants, the methylation levels varied greatly in each genotype, methylation context and tissue type. The flight *elp2-5* plants showed a higher methylation level compared to Col-0 in all methylation contexts in both leaves and roots (Figures 2C,D). The flight *met1-7* methylation levels were lower than Col-0 in the CG context in both leaves and roots. However, *met1-7* showed higher methylation levels than Col-0 in the CHG and CHH contexts in flight leaves while showing similar methylation levels in roots (Figures 2C,D).

In Col-0 the average genome-wide methylation levels within each context were not significantly different between flight and GC plants, for either leaves or roots (Figures 2E,F). However, *met1-7* and *elp2-5* both showed significant increases in methylation levels in spaceflight compared to GCs (Figures 2G–J). The *elp2-5* mutants showed significant differences in the average genome-wide methylation levels between spaceflight and GCs in each organ and all methylation contexts (Figures 2G,H). The *met1-7* mutants shared similar trends as the *elp2-5* mutants in their leaves, however, significant differences between FT and GC were only seen in the CG context of root tissues (Figures 2I,J and Supplementary File 4) includes a detailed breakdown of the distribution of methylation levels, divided into bins ranging from 0 to 100% methylation.

### Spaceflight Changes in DNA Methylation Levels Were Associated With Protein-Coding Gene Regions

**FIGURE 3:**
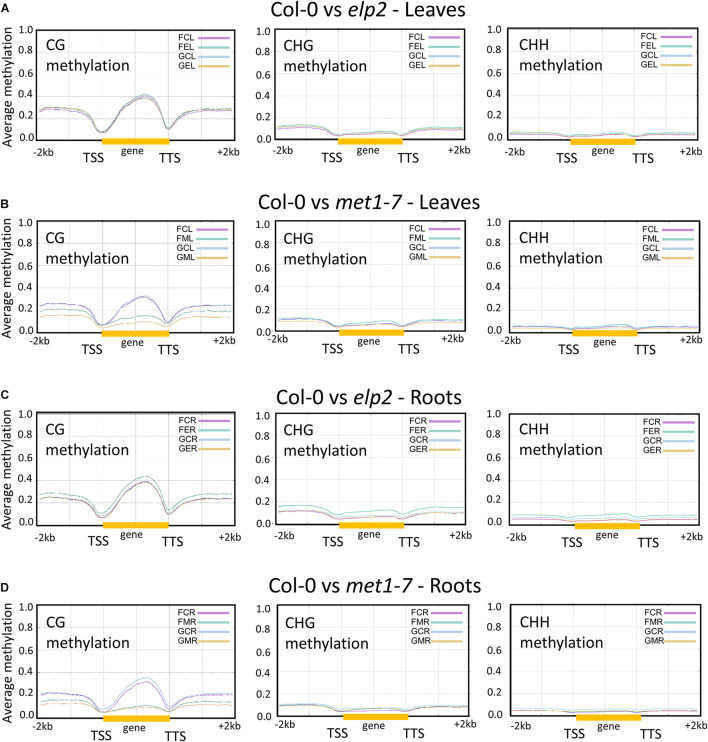
Pairwise comparison of the average methylation levels across protein-coding genes between the genotypes in CG, CHG, and CHH contexts. Gene bodies from transcription start site (TSS) to transcription termination site (TTS; highlighted by the yellow bar) as well as the flanking upstream 2 kb and downstream 2 kb are shown. Each pairwise comparison between genotypes and environment is denoted by a three-letter code. The first letter denotes the environment [flight, (F) and ground (G)], the second letter denotes the genotype [Col-0 (C), *elp2-5* (E), and *met1-7* (M)], and the third letter denotes the specific tissue [leaves (L) and roots (R)]. The *y* axes indicate the average methylation levels in each of the CG, CHG, and CHH contexts. A pairwise comparison between the various genotypes are depicted here Col-0 vs elp2-5 leaves **(A)** and roots **(C)**, along with Col-0 vs met1-7 leaves **(B)** and roots **(D)**.

Changes in DNA methylation induced by spaceflight associated with protein-coding and flanking genic regions [2 kb upstream from the transcription start site (TSS), gene body (gold bar), and 2 kb downstream from transcription termination site] are shown in Figure 3. Pairwise comparison between each methylation mutant and the wild type control are depicted using plot lines color-coded to indicate the environmental conditions [flight, (**F**) and ground (**G**)], the genotype [Col-0 (**C**), *elp2-5* (**E**), and *met1-7* (**M**)] and the tissue type [leaves (**L**) and roots (**R**)], as indicated in the upper right legend of each comparison plot. Thus, the pink line in Figure 3A, **FCL**, reflects the percentage of average methylation for flight, Col-0 leaves across a 4 kb protein-coding region of the genome.

The *elp2-5* and *met1-7* lines differed in their methylation levels across genic regions compared to Col-0. In leaves, Col-0 and *elp2-5* showed similar CG, CHG, and CHH methylation levels across genic regions associated with protein-coding for both spaceflight and GC environments, as seen by the almost overlapping traces in Figure 3A. However, Col-0 and *met1-7* leaves demonstrated notable differences in the CG methylation context. The *met1-7* mutants had lower average CG methylation levels compared to Col-0 across all genic regions, with a pronounced difference within the gene body region (gold bar in graph). In spaceflight, *met1-7* leaves have a higher methylation level across all genic regions when compared to the GCs (Figure 3B).

The *elp2-5* roots showed higher spaceflight-associated CG, CHG, and CHH methylation levels across all genic regions compared to Col-0 (Figure 3C), but there were differences among the contexts with respect to the methylation levels for the other genotypes. In the CG methylation context, the GC Col-0 roots, GC *elp2-5* roots, and flight Col-0 roots all had similar methylation levels, whereas flight *elp2-5* roots showed increased methylation. In the CHG and CHH context, flight Col-0 roots had a slightly lower average methylation level across all genic regions when compared to GCs.

The *met1-7* roots demonstrated noticeable differences in the CG methylation context, with the most pronounced difference within the gene body region (Figure 3D). Flight Col-0 roots had a lower CG methylation level across the gene body regions when compared to the GCs. The flight *met1-7* roots had a higher CG methylation level across flanking upstream and downstream genic regions compared to their GCs. In the CHG methylation contexts, there were no obvious differences between Col-0 and *met1-7* genotypes in either flight or GCs. In the CHH context, flight Col-0 roots, GC *met1-7* roots, and flight *met1-7* roots had similar methylation levels, levels that were slightly lower than GC Col-0 roots (Figure 3D).

### Spaceflight Altered the Distribution and Direction of DmCs in *elp2-5* and *met1-7* Leaves and Roots

**FIGURE 4:**
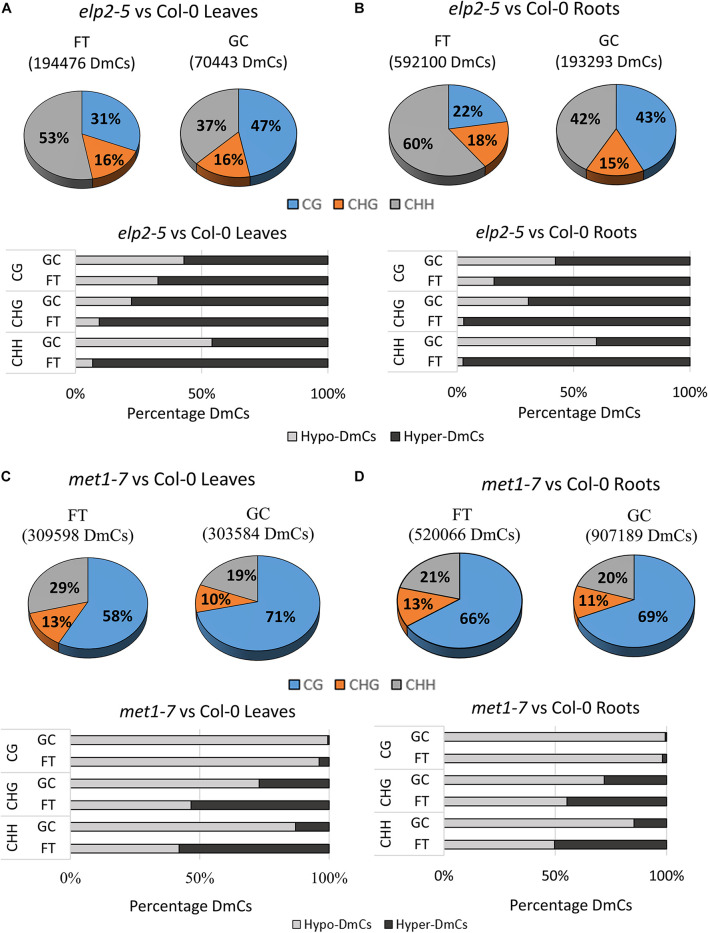
Differentially methylated cytosines (DmCs) of leaves and roots from each genotype in response to spaceflight. **(A)** Breakdown of each methylation context for DmCs in leaves and roots of Col-0, *elp2*, and *met1-7* in spaceflight compared to ground control. **(B)** Total number of DmCs broken down in each context for all three genotypes in leaves and roots. **(C)** Breakdown of the methylation direction of total leaf DmCs in each context – Hypomethylation (Hypo) gray and Hypermethylation (Hyper) black. **(D)** Breakdown of the methylation direction of total root DmCs in each context.

The distribution of cytosines differentially methylated by spaceflight within each methylation context in both leaves and roots was assessed for each mutant relative to the wild-type genotype. DmCs were identified as those cytosines with a differential methylation that was statistically significant to *p* < 0.01 in each comparison and context (Figure 4). The percentage of DmCs was also mapped to different genomic regions for each methylation context. The percentage maps of Upstream (2 kb upstream of the TSS), UTRs, Exons, Introns, Downstream (2 kb downstream of the polyadenylation site), transposable element, Intergenic, and Pseudogenes are shown for each genotype in the flight vs ground comparisons of leaves and roots (Supplementary Figure 3).

In spaceflight, the percentage of DmCs of *elp2-5* relative to Col-0 in leaves is highest in the CHH context (53%; Figure 4A), whereas in the GCs the percentage was highest in the CG context (47%). Similarly, spaceflight roots (Figure 4B) had a higher percentage of DmCs in the CHH context (60%), while in GC roots the majority of the DmCs were divided about equally between CHH (42%) and CG (43%) contexts. In all cases, the percentages of DmCs in the CHG context in the *elp2-5* plants changed very little among organs and environments (15–18%). Most of the *elp2-5* DmCs were hypermethylated (Figures 4A,B). In the *met1-7* and Col-0 comparison, the majority of the DmCs were in the CG context in both leaves and roots, and both organs had lower percentages of CG DmCs in FT than in GC (Figures 4C,D). In leaves, this decrease in the percentage of CG DmCs in FT compared to GC was larger in leaves (13%) compared to roots (3%). In addition, there was a 10% increase in the percentage of CHH DmCs in FT (29%) compared to GC (19%) in the leaves (Figure 4C). In roots, there was only a 1% increase in FT compared to GC (Figure 4D). As was seen in the *elp2-5* plants, the percentage of CHG DmCs changed very little among organs and environments (10–13%). Most of the *met1-7* DmCs were hypomethylated (Figures 4C,D).

**FIGURE 5:**
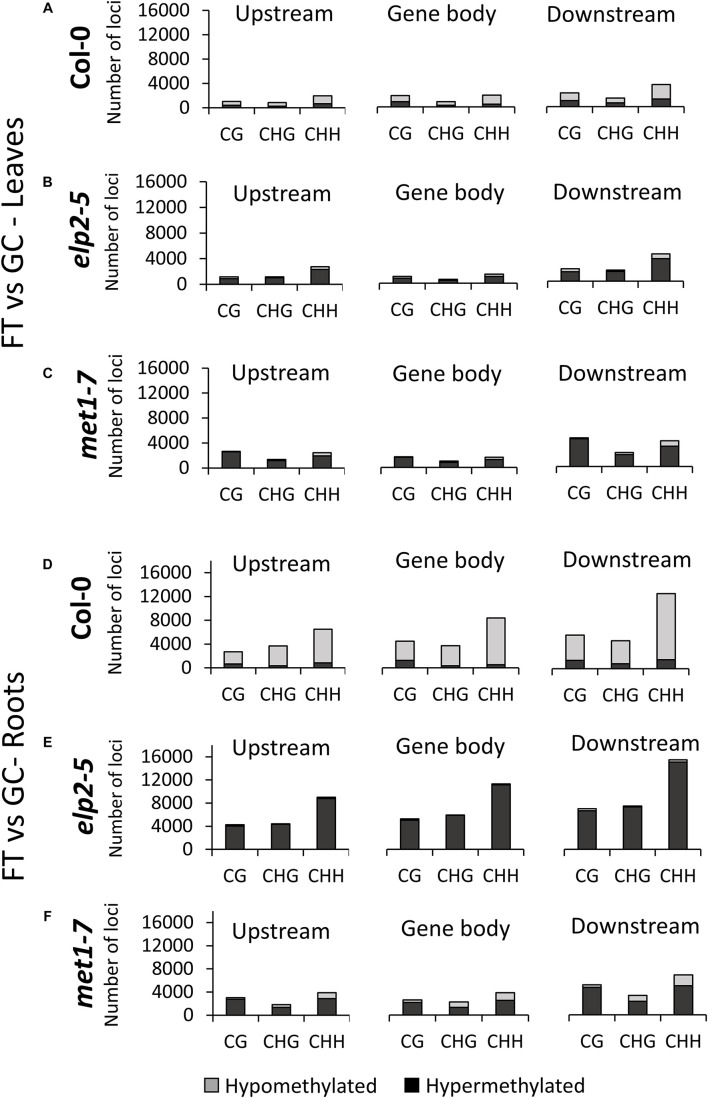
Methylation context breakdown of DmCs mapped to different genic features in spaceflight vs ground comparisons. Leaf and Root DMCs of Col-0 **(A,D)** *elp2-5* **(B,E)**, and *met1-7* **(C,F)** in response to spaceflight are mapped to genic features such as; upstream (2,000 bp upstream of the transcription start site), gene body (exons, introns, and both 5′ and 3′ UTRs), and downstream (2,000 bp downstream of the polyadenylation site). The corresponding methylation direction in each context is also illustrated – Hypomethylation (Hypo) gray and Hypermethylation (Hyper) black.

The distribution of gene-related DmCs in response to spaceflight was genotype- and organ-specific (Figure 5). In Col-0 leaves, DmCs were primarily hypomethylated. In the upstream and downstream flanking regions, the CHH context predominated, but within the gene body the CHH and CG contexts were about equally represented, and CGH methylation the least represented (Figure 5A). In contrast, in both *elp2-5* and *met1-7* leaves the DmCs were primarily hypermethylated (Figures 5B,C). In *elp2-5* leaves methylation in the CHH context predominated in loci within all genic regions (Figure 5B). In *met1-7* leaves the CG and CHH contexts were about equally represented in all genic regions (Figure 5C). In Col-0 roots, DmCs were again primarily hypomethylated, but unlike in leaves, the CHH context predominated within all genic regions (Figure 5D). As with leaves, the DmCs in both *elp2-5* and *met1-7* roots were predominantly hypermethylated (Figures 5E,F). In *elp2-5* roots, methylation of DmCs in the CHH context again predominated in loci within all genic regions, and loci with CG and CHG methylation were about equally distributed in each genic region (Figure 5E). In *met1-7* roots, DmCs with methylation in the CHH contexts were slightly more abundant across all genic regions, but methylation in all contexts was higher in the downstream genic region (Figure 5F).

**FIGURE 6:**
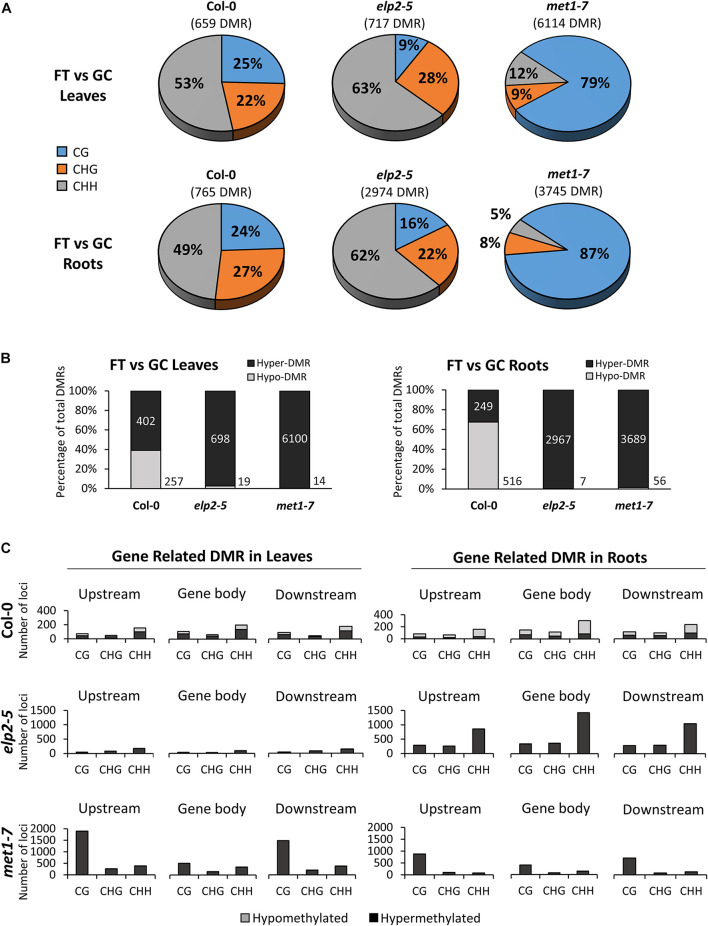
Differentially methylated regions (DMRs) of leaves and roots from each genotype in response to spaceflight. **(A)** Breakdown of each methylation context for DMRs in leaves and roots of Col-0, *elp2-5*, and *met1-7* in spaceflight compared to ground control. **(B)** Breakdown of the methylation direction of total leaf/root DMRs in each genotype in response to spaceflight – Hypomethylation (Hypo) gray and Hypermethylation (Hyper) black. **(C)** Distribution of leaf and root gene-related DMRs of Col-0, *elp2-5*, and *met1-7* in response to spaceflight. DMRs are mapped to genic features such as; upstream (2,000 bp upstream of the transcription start site), gene body (exons, introns, and both 5′ and 3′ UTRs), and downstream (2,000 bp downstream of the polyadenylation site). The corresponding methylation direction in each context is also illustrated – Hypomethylation (Hypo) gray and Hypermethylation (Hyper) black.

Each genotype displayed distinct, organ-specific distributions of DMRs between spaceflight and the GCs (Figure 6). DMRs were determined by comparing the average methylation levels within 100 bp windows between FT and GC, and regions with a statistically significant difference (*p* < 0.01) were used in the analysis. In the Col-0 spaceflight response, 659 DMRs were detected in leaves, and 765 DMRs in roots. In both Col-0 tissues, an average of 51% of the DMRs were found within the CHH context, whereas the other half of the DMR were evenly distributed between the CHG and CG contexts at 24.5% each. In *elp2-5*, there were 717 DMRs in leaves and 2,974 DMRs in roots (Figure 6A). In both *elp2-5* tissues, an average of 63% of the DMRs were found in the CHH context, which was 12% more than Col-0. DMRs in the CHG context were distributed similarly to that of Col-0 at an average of 25%, however, DMRs in the CG context were at an average of 12.5% which was about half that of Col-0. In *met1-7*, there were 6,114 DMRs in leaves and 3,745 DMRs in roots. The majority of the DMRs in both tissues were in the CG context at an average of 83%. DMRs in the CHG context in both tissues were at an average of 8.5% whereas DMRs in the CHH context averaged 12% in the leaves and 5% in the roots (Figure 6A).

The spaceflight-associated DMRs in Col-0 were distributed between hypo and hypermethylation, while hypomethylated DMRs predominated in the mutants. In Col-0 leaves, 39% of the DMRs were hypomethylated and 61% hypermethylated, whereas in Col-0 roots, 67% of the DMRs were hypomethylated and 33% were hypermethylated. However, in both *elp2-5* and *met1-7* leaves and roots, DMRs were predominantly hypermethylated ranging from 97 to 99% (Figure 6B). Gene-related DMRs (Figure 6C) reflected organ-specific responses to spaceflight in all genotypes, but the largest difference in the number of DMR loci between leaves and roots was in *elp2-5*. In Col-0 leaves and roots, the number of DMRs in all genic regions and context was less than 400, with the highest number of DMRs found within the CHH context. In gene-related DMRs in Col-0 leaves hypo- and hypermethylation were fairly evenly distributed across all contexts, whereas in Col-0 roots, gene-related DMRs were mostly hypomethylated (70%). In Col-0 leaves and roots, gene-related DMRs were most abundant in the gene body region. In contrast to wild-type Col-0, the total gene-related DMRs in *elp2-5* were substantially higher in the roots compared to the leaves. In *elp2-5* roots, DMRs were primarily in the CHH context and were predominantly hypermethylated in all genic regions. The highest number of DMRs in the *elp2-5* roots were within the gene body region. In *met1-7*, DMRs were found at a higher number in the leaves compared to the roots. DMRs in both leaves and roots were predominantly hypermethylated and were distributed primarily in the CG context. In *met1-7* leaves, the highest number of DMRs were located upstream of the TSS (Figure 6C).

### Spaceflight Affected More Genes in the Roots of *elp2-5*, and More Genes in the Leaves of *met1-7*

**FIGURE 7:**
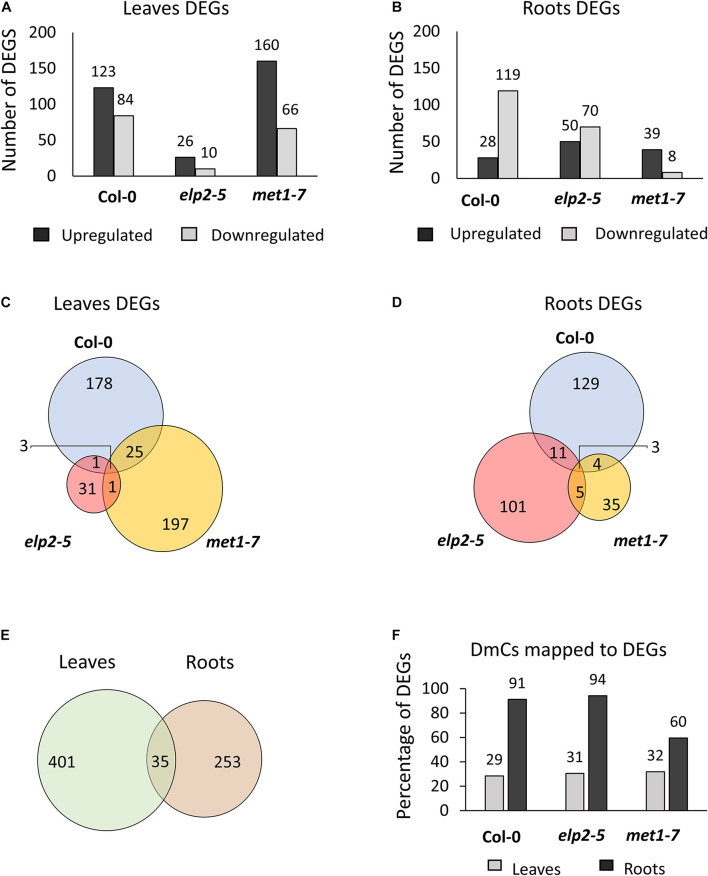
Analysis of differential gene expression in response to spaceflight and the relationship between DmCs. Bar graph showing the number of differentially expressed genes (DEGs) in response to spaceflight (spaceflight compared to ground control) of each genotype in **(A)** leaves or **(B)** roots. Black bars indicate the number of up-regulated genes whereas gray bars indicate the number of down-regulated genes. Venn diagrams showing the DEGs that overlap between each genotype in response to spaceflight in both **(C)** leaves and **(D)** roots. **(E)** Venn diagram of the DEGs that overlap in leaves and roots. **(F)** Bar graph showing the percentage of DmCs that mapped to DEGs in leaves (gray bar) and in roots (black bars).

Spaceflight associated differential gene expression was genotype- and organ-specific; in *elp2-5* more genes were affected in roots, while in *met1-7* more genes were affected in leaves (Figure 7). Transcripts showing at least a twofold change (−1 ≤ log_2_ FC ≥ 1) with an FDR value of < 0.05 were identified as DEGs. PCAs of each type of tissue were performed individually, as leaves and roots have widely different patterns of gene expression (Paul et al., 2013; Zhou et al., 2019). The PCA plots of leaves and roots showed different grouping of *elp2-5* samples along with the components compared to Col-0 and *met1-7* (Supplementary File 2 and Supplementary Figure 1). In the comparison of spaceflight to ground leaves, Col-0 had a total of 207 (123 up-regulated and 84 down-regulated) DEGs, *elp2-5* had 36 (26 up-regulated and 10 down-regulated) DEGs, and *met1-7* had 226 (160 up-regulated and 66 down-regulated) DEGs (Figure 7A). In roots, a total of 147 (28 up-regulated and 119 down-regulated), 120 (50 up-regulated and 70 down-regulated), and 47 (39 up-regulated and 8 down-regulated) DEGs were found in Col-0, *elp2-5*, and *met1-7*, respectively (Figure 7B). Only a few DEGs overlapped between the genotypes in both leaf and root tissues (Figures 7C,D). In leaves, the majority of DEGs were unique to Col-0 and *met1-7*, whereas, in roots, the majority of DEGs were unique to Col-0 and *elp2-5* (Figures 7C,D). The patterns of DEGs in all three genotypes were distinctly organ-specific, and of the 436 DEGs in leaves and 288 in roots, only 35 DEGs were common to both sets (Figure 7E). Although there were overall fewer root-specific DEGs, a greater proportion of the root DEGs were also differentially methylated in response to spaceflight compared to the DEGs of leaves. In roots, 82% of the DEGs also exhibited DmCs, whereas in leaves, 31% of the DEGs also mapped to DmCs (Figure 7F and Supplementary File 5).

**FIGURE 8:**
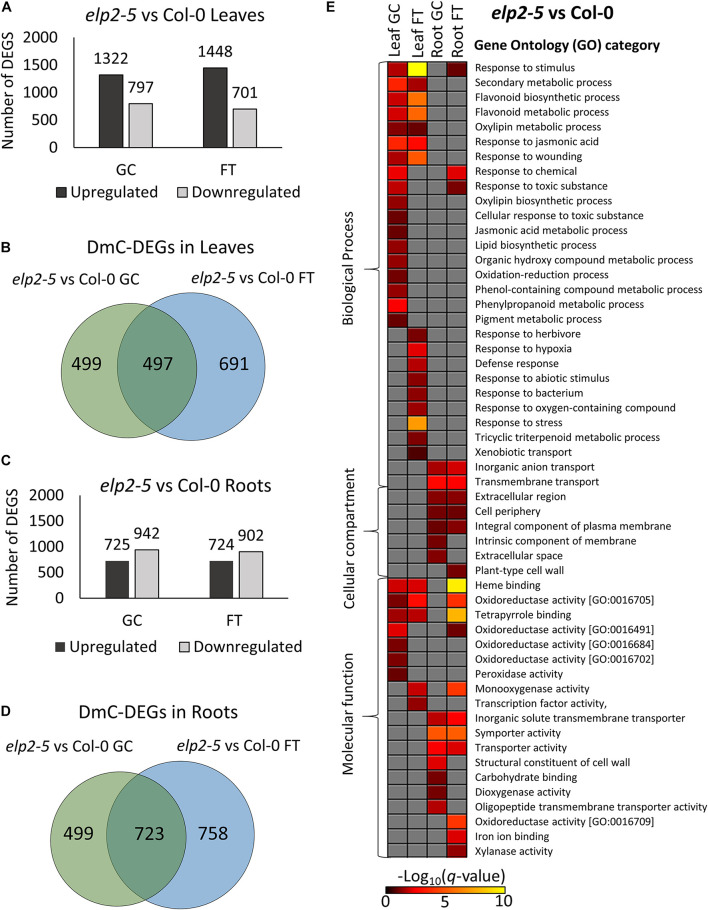
Pairwise comparison of *elp2-5* mutant vs wild-type Col-0 DEGs-DmCs on the ground and in spaceflight. **(A)** Bar graph showing the number of DEGs between *elp2-5* and Col-0 leaves on the ground (GC) and in spaceflight (FT). Black bars indicate the number of up-regulated genes whereas gray bars indicate the number of down-regulated genes. **(B)** Venn diagrams shows the number of DEG-DmCs that overlap between *elp2-5* and Col-0 leaves on the ground and in spaceflight. **(C)** Bar graph showing the number of DEGs between *elp2-5* and Col-0 roots in GC and in FT. Black bars indicate the number of up-regulated genes whereas gray bars indicate the number of down-regulated genes. **(D)** Venn diagrams shows the number of DEG-DmCs that overlap between *elp2-5* and Col-0 roots in GC and in FT. **(E)** Gene ontology (GO) enrichment analyses of DmC-DEG genes in *elp2-5* vs Col-0 comparison of both leaves and roots in each environment (GC and FT). The heatmap shows the GO terms that were enriched within each environment and tissue combination between *elp2-5* and Col-0. The scale bar represents the negative Log_10_ of the *q*-values (corrected *p*-values) from the “test of significance of enrichment” within each list of DmC-DEGs. The *q*-value cutoff was set at 0.05. Higher values in the scaling indicate higher significance of the enrichment.

The composition of DmC-DEGs in *elp2-5* compared to Col-0 was distinctive in each environmental and organ comparison (Figure 8). In leaves, *elp2-5* vs Col-0 on the ground had a total of 2,119 DEGs (1,322 up-regulated and 797 down-regulated) whereas in spaceflight there were a total of 2,149 DEGs (1,448 up-regulated and 701 down-regulated; Figure 8A). Of these, 996 DEGs were also differentially methylated in the GCs, and a total of 1,188 DmC-DEGs were observed in spaceflight (Figure 8B). A total of 497 DmCs-DEGs were shared between both environmental conditions. In roots, the pairwise comparison of *elp2-5* and Col-0 revealed a total of 1,667 (725 up-regulated and 942 down-regulated) DEGs on the ground and 1,626 (724 up-regulated and 902 down-regulated) DEGs in spaceflight (Figure 8C). Of these, 1,222 DEGs on the ground were also associated with DmCs and 1,481 DEGs in spaceflight were associated with DmCs. Among these, 723 DmC-DEGs were shared in both spaceflight and GC conditions (Figure 8D). A visual heat-map summary of the organ-specific distribution of DEGs and the DmC-DEGs for all genotypes is presented in the Supplementary Figure 2, which is annotated in Supplementary File 6.

Multiple metabolic processes appeared to be altered in *elp2-5* compared to Col-0 (Figure 8E). GO analysis of the DmC-DEGs of *elp2-5* compared to Col-0 showed that *elp2-5* was engaged with many processes characteristic of stress responses (Figure 8E). Processes shared as enriched between leaf and root DmC-DEGs included general responses to stimuli, oxidoreductase activities, and heme-binding. Leaf-specific DmC-DEGs were enriched in biosynthetic processes of flavonoids and pigments, jasmonic acid, and wounding responses in both GC and FT. DmC-DEGs in GC leaves were more specifically enriched in phenylpropanoid metabolism and the response to oxidative stress, as well as in further oxidoreducatase and peroxidase activities. DmC-DEGs in FT leaves were enriched with defense and hypoxia responses. DmC-DEGs annotated to the plasma membrane and extracellular structures were enriched among both GC and FT roots, in addition to transport processes and activities. Carbohydrate transport and binding were specifically enriched among root GC DmC-DEGs. Thus, *elp2-5* shows differential expression and methylation of stress and hormone response pathways, as well as transport and metabolic pathways, when compared to the Col-0 wild-type line within each tissue and growth condition.

**FIGURE 9:**
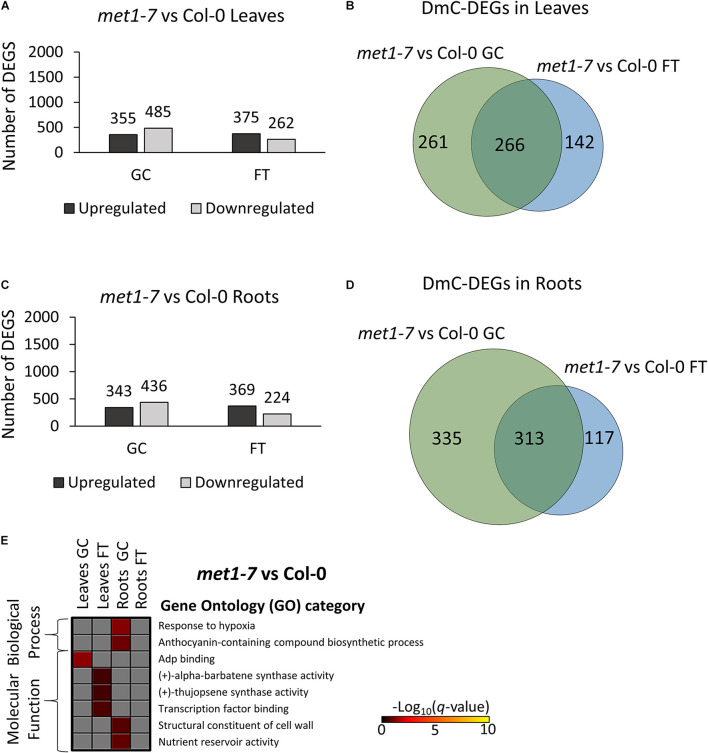
Pairwise comparison of *met1-7* mutant vs wild-type Col-0 DEGs-DmCs on the ground and in spaceflight. **(A)** Bar graph showing the number of DEGs between *met1-7* and Col-0 leaves on the ground (GC) and in spaceflight (FT). Black bars indicate the number of up-regulated genes whereas gray bars indicate the number of down-regulated genes. **(B)** Venn diagrams shows the number of DEG-DmCs that overlap between *met1-7* and Col-0 leaves on the ground and in spaceflight. **(C)** Bar graph showing the number of DEGs between *met1-7* and Col-0 roots in GC and in FT. Black bars indicate the number of up-regulated genes whereas gray bars indicate the number of down-regulated genes. **(D)** Venn diagrams shows the number of DEG-DmCs that overlap between *met1-7* and Col-0 roots in GC and in FT. **(E)** Gene ontology (GO) enrichment analyses of DmC-DEG genes in *met1-7* vs Col-0 comparison of both leaves and roots in each environment (GC and FT). The heatmap shows the GO terms that were enriched within each environment and tissue combination between *met1-7* and Col-0. The scale bar represents the negative Log_10_ of the *q*-values (corrected *p*-values) from the “test of significance of enrichment” within each list of DmC-DEGs. The *q*-value cutoff was set at 0.05. Higher values in the scaling indicate higher significance of the enrichment.

Relative to the Col-0 response, there were substantially more DEGs and DmCs in *elp2-5* (Figure 8) than were seen in *met1-7* (Figure 9). In leaves, *elp2-5* vs Col-0 showed a total of 2,119 (1,322 up-regulated and 797 down-regulated) DEGs in GC, whereas 2,149 (1,448 up-regulated and 701 down-regulated) DEGs were observed in FT (Figure 8A). DmC-DEGs in the *elp2-5* to Col-0 leaves comparison revealed 996 differentially methylated and expressed transcripts on the GC and 1,188 in FT, of which 499 were unique to GC, 691 unique to FT, and 497 were shared among both conditions (Figure 8B). In the roots, the pairwise comparison of *elp2-5* and Col-0 in the GC revealed a total of 1,667 (725 up-regulated and 942 down-regulated) DEGs and a total of 1,626 (724 up-regulated and 902 down-regulated) DEGs in FT (Figure 8C). DmC-DEGs in the *elp2-5* to Col-0 roots comparison revealed a total of 1,222 DmC-DEGs in GC roots and 1,481 DmC-DEGs in FT roots, of which 723 were shared in both conditions (Figure 8D). In leaves, *met1-7* vs Col-0 showed a total of 840 (355 up-regulated and 485 down-regulated) DEGs in GC, whereas in FT 637 (375 up-regulated and 262 down-regulated) DEGs were observed (Figure 9A). DmC-DEGs in the *met1-7* to Col-0 leaves comparison revealed 527 differentially methylated and expressed transcripts on the ground and 408 in flight, of which 261 were unique to GC, 142 unique to FT, and 266 were shared among both conditions (Figure 9B). In the roots, the pairwise comparison of *met1-7* and Col-0 in the GC revealed a total of 779 (343 up-regulated and 436 down-regulated) DEGs and a total of 593 (369 up-regulated and 224 down-regulated) DEGs in spaceflight (Figure 9C). DmC-DEGs in the *met1-7* to Col-0 roots comparison revealed a total of 648 DmC-DEGs in GC roots and 430 DmC-DEGs in FT roots, of which 313 were shared in both conditions (Figure 9D).

Gene ontology analysis of DmC-DEGs between *met1-7* and the wild-type line yielded few term enrichments (Figure 8E). Biological process enrichments associated with hypoxic responses and biosynthesis of anthocyanin-containing compounds were present in GC root DmC-DEGs. Molecular functions of ADP binding, sesquiterpene compound synthase activities, and cell wall constituents, and nutrient reservoir activity were enriched in leaf GC DmC-DEGs, leaf FT DmC-DEGs, and root GC DmC-DEGs, respectively.

**FIGURE 10:**
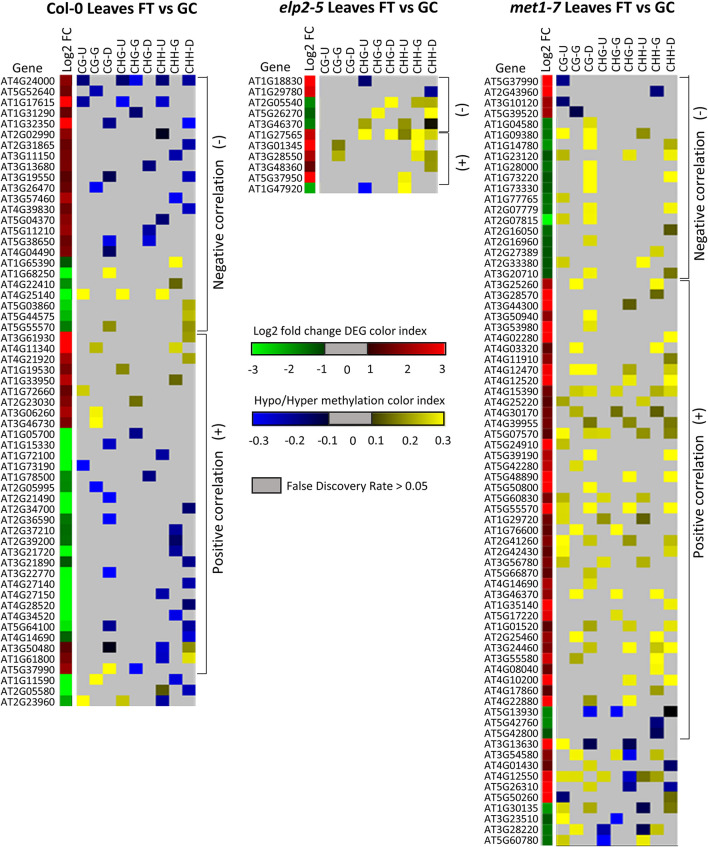
Heatmap of spaceflight (FT) vs ground control (GC) DEGs-DmCs in Col-0, *elp2-5*, and *met1-7* leaves. Heat maps show the Log_2_ (Fold-change) of differential gene expression (red: up-regulated and green: down-regulated) and differential DNA methylation (hypermethylation: yellow and hypomethylation: blue) of Col-0, *elp2-5*, and *met1-7* leaves in FT compared to GC. DmCs for each CG, CHG, and CHH methylation context in each genic region (Gene body: TSS to TTS, upstream: 2 kb from TSS, downstream: 2 kb from TTS) are denoted in the heat maps. The heatmaps show both negative and positive correlations of DEGs with DmCs. Negative correlations (−) are defined when up-regulated genes aligned with hypomethylated DmCs and down-regulated genes aligned with hypermethylated DmCs. The converse relationship is indicative of a positive correlation (+).

**FIGURE 11:**
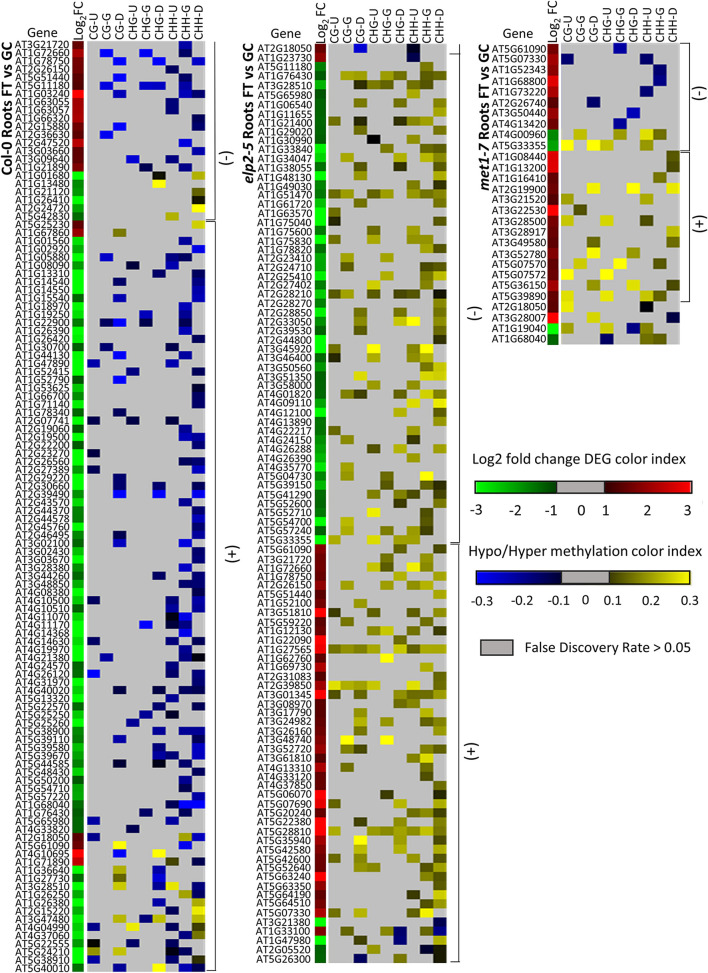
Heatmap of spaceflight (FT) vs ground control (GC) DEGs-DmCs in Col-0, *elp2-5*, and *met1-7* roots. Heat maps show the Log_2_ (Fold-change) of differential gene expression (red: up-regulated and green: down-regulated) and differential DNA methylation (hypermethylation: yellow and hypomethylation: blue) of Col-0, *elp2-5*, and *met1-7* roots in FT compared to GC. DmCs for each CG, CHG, and CHH methylation context in each genic region (Gene body: TSS to TTS, upstream: 2 kb from TSS, downstream: 2 kb from TTS) are denoted in the heat maps. The heatmaps show both negative and positive correlations of DEGs with DmCs. Negative correlations (−) are defined when up-regulated genes aligned with hypomethylated DmCs and down-regulated genes aligned with hypermethylated DmCs. The converse relationship is indicative of a positive correlation (+).

Alignments of DmC-DEGs in each methylation context in the various genic regions were generated for each genotype and depicted as heatmaps (Figure 10). In leaves, a total of 59 DmC-DEGs were observed in wild-type Col-0, while in *elp2-5* and *met1-7*, there were 11 and 72 DmC-DEGs, respectively. Correlations between differential expression and DNA methylation were used to organize the heatmap. The top bracketed sections of the heatmaps show negative correlations where up-regulated genes aligned with hypomethylated DmCs and down-regulated genes aligned with hypermethylated DmCs are grouped. The following bracketed section shows positive correlations where the converse relationship between DEGs and DmCs was highlighted. The unbracketed section at the bottom of the heatmap shows genes that have no distinguishable correlations between DEGs and DmCs. In Col-0 leaves, 41% (24 out of 59) of genes had a negative correlation and 49% (29 out of 59) had a positive correlation. In *elp2-5* leaves, negative and positive correlation were evenly distributed at 45% (5 out of 11), whereas in *met1-7* leaves, 26% (19 out of 72) of genes showed a negative correlation and 60% (43 out of 72) had a positive correlation. In roots, there were a total of 114 DmC-DEGs in Col-0, 101 DmC-DEGs in *elp2-5*, and 28 DmC-DEGs in *met1-7* (Figure 11). 16% (19 out of 114) of the wild-type Col-0 root DmC-DEGs had a negative correlation between gene expression and methylation levels, whereas 66% (75 out of 114) were positively correlated. In *elp2-5* roots, 54% (55 out of 101) of genes showed a negative correlation, and 42% (42 out of 101) showed a positive correlation. In *met1-7* roots, 36% (10 out of 28) of genes showed a negative correlation, and 50% (14 out of 28) showed a positive correlation.

**FIGURE 12:**
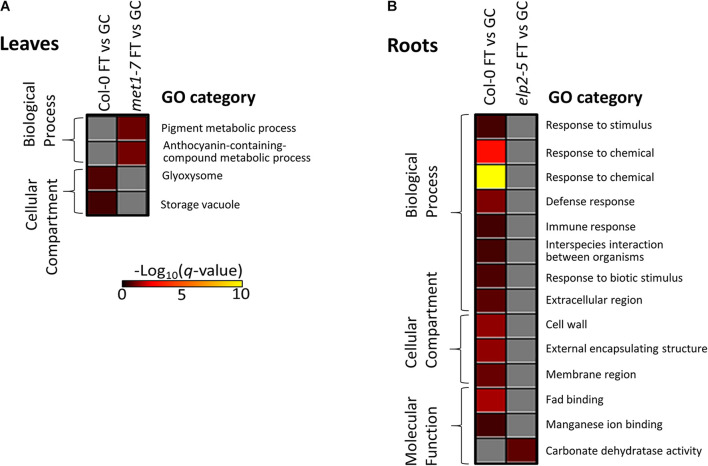
Gene ontology enrichment analyses of spaceflight (FT) vs ground control (GC) DEGs-DmCs in each genotype. **(A)** The heatmap shows the GO terms that were enriched for in the DmC-DEG heatmap (Figure 10) for the leaf tissue in all three genotypes. In leaves, *elp2-5* did not have any enriched GO terms. **(B)** The heatmap shows GO terms that were enriched for in the DmC-DEG heatmap (Figure 11) for the root tissue in all three genotypes. In roots, *met1-7* did not have any enriched GO terms. The scale bar represents the negative Log_10_ of the *q*-values (corrected *p*-values) for the GO terms’ enrichment within each list of DmC-DEGs. The *q*-value cutoff was set at 0.05. Higher values in the scaling indicate higher significance of the enrichment.

Gene ontology analysis of the lists of DmC-DEGs from the FT vs GC contrasts revealed that the *elp2-5* and *met1-7* lines lacked enrichment of differential expression in traditional spaceflight acclimation processes (Figure 12). In Col-0 leaves, DmC-DEGs were enriched in localization to glyoxosomes (Figure 12A). The DmC-DEGs observed in the spaceflight response of *met1-7* leaves were in pathways associated with the metabolism of pigments containing anthocyanins. The enrichments among the Col-0 root FT vs GC DmC-DEGs were primarily associated with defense pathways and responses to hypoxia, as well as the cell wall and membrane nanodomains (Figure 12B and Supplementary Table 1). Col-0 and *elp2-5* roots showed enrichments of molecular function GO terms, involving FAD and manganese ion binding, and carbonate dehydratase activity, respectively. A secondary analysis using all FT vs GC DEGs showed that the analysis of only DmC-DEGs recapitulated the majority of the terms associated with the overall transcriptomic response to spaceflight (data not shown). The GO-associated processes, localizations, and functions represented by these terms are gained or lost from the methylation-sensitive aspect of the spaceflight response dependent on the functionality of ELP2 and MET1.

## Discussion

DNA methylation plays a crucial role in regulating stress responses and physiological adaptation in plants (Boyko and Kovalchuk, 2010; Dowen et al., 2012; Zhang, 2012; Wang et al., 2013; Yu et al., 2013; Garg et al., 2015; Tameshige et al., 2015; Yong-Villalobos et al., 2015; Zhou et al., 2019). Physiological adaptation to spaceflight engages gene expression changes that at least mimic several terrestrial stress responses (Hoson et al., 2002; Gao et al., 2008; Salmi and Roux, 2008; Blancaflor, 2013; Correll et al., 2013; Paul et al., 2013; Zupanska et al., 2013; Ferl et al., 2014; Inglis et al., 2014; Nakashima et al., 2014; Sugimoto et al., 2014; Kwon et al., 2015; Schüler et al., 2015; Zhang et al., 2015; Barker et al., 2017; Choi et al., 2019; Kruse et al., 2020; Supplementary Table 2). In addition, the first whole-genome survey of DNA methylation in wild-type WS Arabidopsis plants grown entirely in the spaceflight environment showed correlated changes in DNA methylation accompanying regulation of DEGs in spaceflight, especially in stress response genes such as those associated with defense responses and ROS signaling (Zhou et al., 2019). However, such correlations do not establish a functional connection between DNA methylation and physiological adaptation to the spaceflight environment. Therefore, two methylation mutant lines, each deficient in separate and distinct functions that affect methylation, were grown on the ISS to assess their response to spaceflight compared to that of wild type. The assays for response to spaceflight were overall growth and morphology, gene expression profiles, and DNA methylation profiles.

The *elp2-5* plants failed to grow normally in spaceflight (Figure 1A). Previous experiments have shown that in the microgravity of spaceflight, plant root growth is altered compared to growth on the ground, yet when presented with directional light, roots generally grow away from the light source and establish a near normal growth pattern, and present a generally healthy-looking morphology (Paul et al., 2012; Zhou et al., 2019). However, the roots of the *elp2-5* plants in spaceflight did not set up this typical spaceflight directionality, as the roots did not navigate away from the germinated seed. The total biomass of the *elp2-5* plants on orbit was similar to that of the GCs (Supplementary Figure 1). Thus, all of the biomass of the *elp2-5* spaceflight plants was concentrated into the area very near the seed, which may accelerate the depletion of nutrients in the immediate vicinity of germination. Nutrient depletion stress is also apparent in the *elp2-5* spaceflight transcriptome. The leaves of the spaceflight *elp2-5* plants appeared to be less expanded, more chlorotic and displayed more reddish pigmentation than the leaves of the GCs (Figure 1C). The *elp2-5* mutants generally exhibit over-accumulation of anthocyanin pigments, which can be further exacerbated by stress (Zhou et al., 2009). This distinct, spaceflight-dependent, morphology for *elp2-5* plants suggests that *elp2-5* plants are physiologically maladapted to spaceflight. While it has been established that ELP2 has a role in root development (e.g., Jia et al., 2015) the spaceflight data suggest that ELP2 may also play a role in gravity perception and root navigation. In terrestrial environments, where gravity can impart a tropic force, plants lacking ELP2 have shorter roots but grow reasonably well (Jia et al., 2015). However, in the absence of gravity, *elp2-5* mutant roots appear to lose the ability to navigate effectively away from the site of the germinating seed.

The *met1-7* plants appeared better able to cope with the absence of gravity than *elp2-5* plants, as their spaceflight root growth patterns were similar to Col-0, with no obvious deleterious morphologies seen on orbit (Figure 1C). The *met1-7* plants produced as much biomass on orbit as in the GCs (Supplementary Figure 1) and established a root growth pattern that was visually typical of spaceflight growth (Paul et al., 2012; Zhou et al., 2019) and similar to the appearance of Col-0 (Figure 1C).

The differential spaceflight morphologies of *elp2-5* and *met1-7* suggest that the DNA methylation events conditioned by MET1 and ELP2 differentially affect spaceflight physiological adaptation and success. Since MET1 and ELP2 represent two different mechanisms that affect methylation, these results suggest that spaceflight might affect the methylation of specific genes more than the genome-wide status of methylation.

The degree of genome-wide methylation was not statistically different between spaceflight and GCs for Col-0, but both *elp2-5* and *met1-7* were generally hypermethylated compared to the GCs (Figure 2 and Supplementary File 1). This result reinforces the conclusion that changes in overall average genome methylation is not a hallmark of spaceflight adaptation in wild-type plants, as this result was also seen in the spaceflight methylome of Arabidopsis cultivar WS (Zhou et al., 2019). However, the hypermethylation of the mutant genomes suggested that the respective contributions of MET1 and ELP2 are important to maintaining an appropriate degree of genome-wide methylation in the physiological adaptation to spaceflight. The degree of DNA methylation within each context in *elp2-5* was significantly increased in response to spaceflight in both leaves and roots (Figures 2G,H) compared to the degree of methylation of these mutants with respect to wild type in the GCs. However, there was a distinction between the two mutant lines in the degree and context of methylation in the respective root genomes. The spaceflight *elp2-5* root genome was significantly hypermethylated in all contexts (CG, CHG, and CHH) compared to the GCs, whereas only CG methylation was elevated in the spaceflight *met1-7* genome (Figures 2D,H,J). Although the contribution of CHG and CHH methylation in Arabidopsis is generally small (about 14%; Niederhuth et al., 2016) it is possible that while genome-wide changes in CG methylation does not impact spaceflight root growth morphology, changes in the CHG and CHH context may contribute to the spaceflight phenotype of *elp2-5* plants.

The degree of spaceflight methylation across protein-coding regions showed organ-specificity among all genotypes. Methylation in the protein coding regions is relevant to gene expression profiles, which are also highly organ specific. Spaceflight induced more CG methylation across all protein-coding regions (upstream, gene body, and downstream) in *met1-7* leaves and only increased methylation in the upstream and downstream regions in roots (Figure 3). Conversely, *elp2-5* methylation was increased across all protein-coding regions and methylation contexts only in the roots in response to spaceflight (Figure 3). The substantial increase in CHH methylation in *elp2-5* roots (Figures 4A,B) suggests that ELP2 plays a dominant role in governing the spaceflight-associated methylation changes in the roots, specifically in the CHH context. This correlates with the root growth morphology exhibited by *elp2-5*.

Col-0, *met1-7*, and *elp2-5* are each different in their spaceflight gene expression and DNA methylation profiles. The profiles of differentially expressed and differentially methylated genes in both *elp2-5* and *met1-7* were unique compared to wild-type Col-0 (Figures 10, 11 and Supplementary Figure 2). The loss of MET1 and ELP2 each had a substantial influence on the profiles of DEGs and differentially methylated genes in response to the spaceflight environment. The types and functions of the genes that are differentially expressed in the *elp2-5* spaceflight plants indicate that the spaceflight plants are under considerable stress compared to GCs. Almost a third of the genes uniquely induced in the spaceflight *elp2-5* leaves are associated with nutrient stress, and most of the rest are genes associated with heat stress, ROS and pathogen responses. These gene classes were also noticeably represented among the *elp2-5* spaceflight root DEGs. These gene expression patterns describe a highly stressed response reflecting the compact growth morphology of the spaceflight *elp2-5* plants. The types and functions of the genes that are differentially expressed in the *met1-7* spaceflight plants suggest that *met1-7* plants were better adjusted to spaceflight than the *elp2-5* plants. As with the *elp2-5* plants, the *met1-7* plants present a number of stress-associated DEGs. However, in contrast to the types of genes expressed by spaceflight *elp2-5* plants, the DEGs in spaceflight *met1-7* plants predominantly functioned in signal transduction, as membrane transporters or transcriptional activators (Supplementary Figure 2 and Supplementary File 6).

While organ-specific differences in the gene expression patterns of various Arabidopsis tissue types (leaves, hypocotyls, roots, and root tips) have been previously reported in spaceflight transcriptomes (Paul et al., 2013, 2017; Zhou et al., 2019), the *met1-7* and *elp2-5* plant lines each demonstrated a different pattern of organ-specific expression and methylation. A large proportion of the differentially methylated and expressed genes in leaves were observed in Col-0 (59 out of 207) and *met1-7* (72 out of 226), whereas in roots a large proportion of differentially methylated and expressed genes were found in Col-0 (114 out of 147) and *elp2-5* (101 out of 120). Only 8% of the leaf DEGs and 12% of the root DEGs overlap between the genotypes, suggesting that unique organ-specific mechanisms were engaged for spaceflight adaptation, with MET1 playing a more important role in leaves and ELP2 having the more important role in roots, in the physiological adaptation to spaceflight.

The relationships between DNA methylation and gene expression in spaceflight were complex and differed between leaves and roots. In wild-type Col-0 leaves, the hypomethylated DmCs and hypermethylated DmCs were evenly distributed across all contexts and all genic regions (Figure 5A). The association of changes in methylation with gene expression showed an even proportion of positive and negative correlation. In *met1-7* leaves, DmCs were predominantly hypermethylated in the CG and CHH contexts of downstream regions, and a larger proportion of positive correlations were observed among DmC-DEGs (Figure 10). In *elp2-5* roots, DmCs were primarily hypermethylated in the CHH context across all genic regions, yet a larger proportion of DmC-DEGs showed a negative correlation (Figure 11). These observations are in contrast with other studies that report a strong negative correlation between promoter CG methylation and gene expression levels (Finnegan et al., 1996; Garg et al., 2015; Jia et al., 2015). Further investigations may clarify the relationship between leaf spaceflight transcript abundance and methylation in different contexts and genic regions. However, 80% of DEGs (across all genotypes) were also differentially methylated in roots. In leaves, that average was only 30% (Figure 7F). These data suggest that DNA methylation plays a significantly larger role in regulating the genes associated with the spaceflight response in roots than in leaves.

The genes differentially regulated by *elp2-5* and *met1-7* in spaceflight were from distinctly different metabolic processes than those regulated in Col-0 in response to spaceflight. Functional analyses of DmC-DEGs showed sharp contrasts among *elp2-5* and *met1-7* with the Col-0 wild-type. The mutant e*lp2-5* line showed differential methylation and expression of gene classes traditionally associated with the spaceflight response and other abiotic stresses, such as metabolic processes and defense, hormone, and hypoxic responses across tissue types (Figure 8E; Cramer et al., 2011). The mutant *met1-7* line, conversely, demonstrated relatively few GO term enrichments, indicating that DmC-DEGs tended to result from largely untargeted changes in methylation (Figure 9E). ELP2 regulates root growth and development, and *elp2* loss of function mutants display shorter roots, and this phenotype may be linked to the root-specific involvement of DmC-DEGs associated with ion homeostasis, transport processes, and extracellular localization observed in this study (Figures 1C, 8E; Jia et al., 2015). The spaceflight-associated processes that are differentially represented in *elp2-5* are also among the primary classes enriched in DmC-DEGs involved in the spaceflight acclimation of Col-0 (Figures 8E, 12). These data suggest that the spaceflight response works more directly through DNA methylation mechanisms and other mechanisms regulated by ELP2 than through the maintenance mechanisms represented by MET1.

DNA methylation profiles within a genome are dynamic and complex, yet integral to plant growth, development, and stress responses (reviewed in: Bartels et al., 2018). The response to spaceflight includes differential DNA methylation in a manner that is similar to known terrestrial stress responses, particularly those associated with pathogen attack or harsh environments. Supplementary Table 2 provides a comparison of these spaceflight-associated DmC-DEGs with those from several terrestrial studies of environmental stress responses (Yong-Villalobos et al., 2016; Hewezi et al., 2017, 2018; Stassen et al., 2018; Korotko et al., 2021). This correlation strongly suggests that the spaceflight response, though novel, utilizes a range of mechanistic approaches that are typically employed during terrestrial environmental stress. While direct methylation mechanisms represented by MET1 certainly affect the spaceflight response in terms of gene expression profiles, the indirect DNA demethylation/methylation mechanisms associated with ELP2 have a more profound role than MET1 in the spaceflight response.

## Conclusion

Genetic factors that influence genome DNA methylation directly impact physiological adaptation to spaceflight, affecting overall growth in space as well as the specifics of the spaceflight gene response profile. Genome methylation is therefore important for spaceflight responses in much the same way as it is important for adaptation to terrestrial stresses. In particular, processes regulated by ELP2 appear critical for proper root directional development in spaceflight. The remodeling of the Arabidopsis methylome in spaceflight, together with the negative outcomes of interfering with DNA methylation, suggests that epigenetic marking is a fundamental part of environmental responses, even during novel environmental stresses outside of the evolutionary history of plants.

## Data Availability Statement

The original contributions presented in the study are publicly available. This data can be found here: The RNASeq and bisulfite-seq data reported in this article have been deposited with NCBI at Gene Expression Omnibus (GEO) repository under the super series accession number GSE118503 (GSE118483 for Bisulfite-seq and GSE118502 for RNA seq).

## Author Contributions

A-LP and RF contributed equally and were responsible for the overall experimental design, execution of the spaceflight experiments, final data evaluations, and writing of the manuscript. NH performed RNA extraction for RNAseq analysis, data analysis, and had a substantial role in initial manuscript and figure development. BC conducted the GO analysis and contributed to the final editing and figure development. All authors contributed to the writing.

## Conflict of Interest

The authors declare that the research was conducted in the absence of any commercial or financial relationships that could be construed as a potential conflict of interest.

## Publisher’s Note

All claims expressed in this article are solely those of the authors and do not necessarily represent those of their affiliated organizations, or those of the publisher, the editors and the reviewers. Any product that may be evaluated in this article, or claim that may be made by its manufacturer, is not guaranteed or endorsed by the publisher.

## Footnotes

1
https://biotech.ufl.edu/next-gen-dna/

2
https://biotech.ufl.edu/gene-expression-genotyping/

3
https://biotech.ufl.edu/bioinformatics/

## References

[B1] Abe H. Yamaguchi-Shinozaki K. Urao T. Iwasaki T. Hosokawa D. Shinozaki K. (1997). Role of arabidopsis MYC and MYB homologs in drought- and abscisic acid-regulated gene expression. *Plant Cell* 9 1859–1868. 9368419 10.1105/tpc.9.10.1859 PMC157027

[B2] Akhter Z. Bi Z. Ali K. Sun C. Fiaz S. Haider F. U. (2021). In Response to Abiotic Stress, DNA Methylation Confers EpiGenetic Changes in Plants. *Plants* 10:1096. 10.3390/plants10061096 34070712 PMC8227271

[B3] Andrews S. (2010). *FastQC**: A quality control tool for high-throughput sequence data [Online].* Available online at: https://www.bioinformatics.babraham.ac.uk/projects/fastqc/ (accessed January 29, 2018).

[B4] Andrews S. (2018). *Babraham Bioinformatics - FastQC A Quality Control tool for High Throughput Sequence Data [Online].* Available online at: https://www.bioinformatics.babraham.ac.uk/projects/fastqc/ (accessed January 29, 2018).

[B5] Angelos E. Ko D. K. Zemelis-Durfee S. Brandizzi F. (2021). Relevance of the Unfolded Protein Response to Spaceflight-Induced Transcriptional Reprogramming in Arabidopsis. *Astrobiology* 21 367–380. 10.1089/ast.2020.2313 33325797 PMC7987364

[B6] Ashapkin V. V. Kutueva L. I. Aleksandrushkina N. I. Vanyushin B. F. (2020). Epigenetic Mechanisms of Plant Adaptation to Biotic and Abiotic Stresses. *Int. J. Mol. Sci.* 21:E7457. 10.3390/ijms21207457 33050358 PMC7589735

[B7] Barker R. Lombardino J. Rasmussen K. Gilroy S. (2020). Test of Arabidopsis Space Transcriptome: a Discovery Environment to Explore Multiple Plant Biology Spaceflight Experiments. *Front. Plant Sci.* 11:147. 10.3389/fpls.2020.00147 32265943 PMC7076552

[B8] Barker R. Swanson S. Gilroy S. (2017). *The TOAST database: a data visualization tool for astrobotany research.* Available online at: https://astrobotany.com/toast/

[B9] Bartels A. Han Q. Nair P. Stacey L. Gaynier H. Mosley M. (2018). Dynamic DNA Methylation in Plant Growth and Development. *Int. J. Mol. Sci.* 19:2144. 10.3390/ijms19072144 30041459 PMC6073778

[B10] Beyrne C. C. Iusem N. D. Gonzalez R. M. (2019). Effect of Salt Stress on Cytosine Methylation within GL2, An Arabidopsis thaliana Gene Involved in Root Epidermal Cell Differentiation. Absence of Inheritance in the Unstressed Progeny. *Int. J. Mol. Sci.* 20:E4446. 10.3390/ijms20184446 31509941 PMC6769687

[B11] Bilichak A. Ilnystkyy Y. Hollunder J. Kovalchuk I. (2012). The Progeny of Arabidopsis thaliana Plants Exposed to Salt Exhibit Changes in DNA Methylation, Histone Modifications and Gene Expression. *PLoS One* 7:e30515. 10.1371/journal.pone.0030515 22291972 PMC3264603

[B12] Blancaflor E. B. (2013). Regulation of plant gravity sensing and signaling by the actin cytoskeleton. *Am. J. Bot.* 100 143–152. 10.3732/ajb.1200283 23002165

[B13] Bolger A. M. Lohse M. Usadel B. (2014). Trimmomatic: a flexible trimmer for Illumina sequence data. *Bioinformatics* 30 2114–2120. 10.1093/bioinformatics/btu170 24695404 PMC4103590

[B14] Boyko A. Kovalchuk I. (2010). Transgenerational response to stress in Arabidopsis thaliana. *Plant Signal. Behav.* 5 995–998. 10.4161/psb.5.8.12227 20724818 PMC3115178

[B15] Califar B. Sng N. J. Zupanska A. Paul A.-L. Ferl R. J. (2020). Root Skewing-Associated Genes Impact the Spaceflight Response of Arabidopsis thaliana. *Front. Plant Sci.* 11:239. 10.3389/fpls.2020.00239 32194611 PMC7064724

[B16] Chevalier D. Batoux M. Fulton L. Pfister K. Yadav R. K. Schellenberg M. (2005). STRUBBELIG defines a receptor kinase-mediated signaling pathway regulating organ development in Arabidopsis. *Proc. Natl. Acad. Sci. U. S. A.* 102 9074–9079. 10.1073/pnas.0503526102 15951420 PMC1157047

[B17] Choi W. G. Barker R. J. Kim S. H. Swanson S. J. Gilroy S. (2019). Variation in the transcriptome of different ecotypes of Arabidopsis thaliana reveals signatures of oxidative stress in plant responses to spaceflight. *Am. J. Bot.* 106 123–136. 10.1002/ajb2.1223 30644539

[B18] Colaneri A. C. Jones A. M. (2013). Genome-Wide Quantitative Identification of DNA Differentially Methylated Sites in Arabidopsis Seedlings Growing at Different Water Potential. *PLoS One* 8:e59878. 10.1371/journal.pone.0059878 23577076 PMC3620116

[B19] Correll M. J. Pyle T. P. Millar K. D. L. Sun Y. Yao J. Edelmann R. E. (2013). Transcriptome analyses of Arabidopsis thaliana seedlings grown in space: implications for gravity-responsive genes. *Planta* 238 519–533. 10.1007/s00425-013-1909-x 23771594

[B20] Cramer G. R. Urano K. Delrot S. Pezzotti M. Shinozaki K. (2011). Effects of abiotic stress on plants: a systems biology perspective. *BMC Plant Biol.* 11:163. 10.1186/1471-2229-11-163 22094046 PMC3252258

[B21] DeFraia C. T. Zhang X. Mou Z. (2010). Elongator subunit 2 is an accelerator of immune responses in Arabidopsis thaliana. *Plant J.* 64 511–523. 10.1111/j.1365-313X.2010.04345.x 20807211

[B22] Dhami N. Cazzonelli C. I. (2020). Prolonged cold exposure to Arabidopsis juvenile seedlings extends vegetative growth and increases the number of shoot branches. *Plant Signal. Behav.* 15:1789320. 10.1080/15592324.2020.1789320 32631114 PMC8550187

[B23] Ding Y. Mou Z. (2015). Elongator and its epigenetic role in plant development and responses to abiotic and biotic stresses. *Front. Plant Sci.* 6:296. 10.3389/fpls.2015.00296 25972888 PMC4413731

[B24] Dowen R. H. Pelizzola M. Schmitz R. J. Lister R. Dowen J. M. Nery J. R. (2012). Widespread dynamic DNA methylation in response to biotic stress. *Proc. Natl. Acad. Sci. U. S. A.* 109 E2183–E2191. 10.1073/pnas.1209329109 22733782 PMC3420206

[B25] Ewels P. Magnusson M. Lundin S. Käller M. (2016). MultiQC: summarize analysis results for multiple tools and samples in a single report. *Bioinformatics* 32 3047–3048. 10.1093/bioinformatics/btw354 27312411 PMC5039924

[B26] Ferl R. J. Koh J. Denison F. Paul A.-L. (2014). Spaceflight Induces Specific Alterations in the Proteomes of Arabidopsis. *Astrobiology* 15 32–56. 10.1089/ast.2014.1210 25517942 PMC4290804

[B27] Ferl R. J. Paul A.-L. (2016). The effect of spaceflight on the gravity-sensing auxin gradient of roots: GFP reporter gene microscopy on orbit. *NPJ Microgravity* 2:15023. 10.1038/npjmgrav.2015.23 28725721 PMC5515520

[B28] Finnegan E. J. Peacock W. J. Dennis E. S. (1996). Reduced DNA methylation in Arabidopsis thaliana results in abnormal plant development. *Proc. Natl. Acad. Sci. U. S. A.* 93 8449–8454. 10.1073/pnas.93.16.8449 8710891 PMC38691

[B29] Fitzgerald C. Barker R. Choi W.-G. Swanson S. Stephens S. Huber C. (2016). Development of Equipment that Uses Far-Red Light to Impose Seed Dormancy in Arabidopsis for Spaceflight. *Gravit. Space Res.* 4 8–19. 10.2478/gsr-2016-0008

[B30] Gao X. Nagawa S. Wang G. Yang Z. (2008). Cell Polarity Signaling: focus on Polar Auxin Transport. *Mol. Plant* 1 899–909. 10.1093/mp/ssn069 19825591 PMC2902905

[B31] Garg R. Chevala V. V. S. N. Shankar R. Jain M. (2015). Divergent DNA methylation patterns associated with gene expression in rice cultivars with contrasting drought and salinity stress response. *Sci. Rep.* 5:14922. 10.1038/srep14922 26449881 PMC4598828

[B32] Glatt S. Muller C. W. (2013). Structural insights into Elongator function. *Curr. Opin. Struct. Biol.* 23 235–242. 10.1016/j.sbi.2013.02.009 23510783

[B33] Guo H. Y. Gao Z. Q. Zhang H. Wei Y. Xu J. H. Wang W. Y. (2013). Purification, crystallization and preliminary crystallographic analysis of the 23S rRNA methyltransferase RlmM (Cm2498) from *Escherichia coli*. *Acta Crystallogr. Sect. F Struct. Biol. Cryst. Commun.* 69 640–642. 10.1107/S1744309113006611 23722841 PMC3668582

[B34] Herranz R. Vandenbrink J. P. Villacampa A. Manzano A. Poehlman W. L. Feltus F. A. (2019). RNAseq Analysis of the Response of Arabidopsis thaliana to Fractional Gravity Under Blue-Light Stimulation During Spaceflight. *Front. Plant Sci.* 10:1529. 10.3389/fpls.2019.01529 31850027 PMC6889863

[B35] Hewezi T. Lane T. Piya S. Rambani A. Rice J. H. Staton M. (2017). Cyst Nematode Parasitism Induces Dynamic Changes in the Root Epigenome. *Plant Physiol.* 174 405–420. 10.1104/pp.16.01948 28298479 PMC5411145

[B36] Hewezi T. Pantalone V. Bennett M. Neal Stewart C. Jr. Burch-Smith T. M. (2018). Phytopathogen-induced changes to plant methylomes. *Plant Cell Rep.* 37 17–23. 10.1007/s00299-017-2188-y 28756583

[B37] Hirayama T. Shinozaki K. (2010). Research on plant abiotic stress responses in the post-genome era: past, present and future. *Plant J.* 61 1041–1052. 10.1111/j.1365-313X.2010.04124.x 20409277

[B38] Hoson T. Soga K. Mori R. Saiki M. Nakamura Y. Wakabayashi K. (2002). Stimulation of Elongation Growth and Cell Wall Loosening in Rice Coleoptiles under Microgravity Conditions in Space. *Plant Cell Physiol.* 43 1067–1071. 10.1093/pcp/pcf126 12354926

[B39] Hsieh W. P. Hsieh H. L. Wu S. H. (2012). Arabidopsis bZIP16 transcription factor integrates light and hormone signaling pathways to regulate early seedling development. *Plant Cell* 24 3997–4011. 10.1105/tpc.112.105478 23104829 PMC3517232

[B40] Huang H. Ullah F. Zhou D.-X. Yi M. Zhao Y. (2019). Mechanisms of ROS Regulation of Plant Development and Stress Responses. *Front. Plant Sci.* 10:800. 10.3389/fpls.2019.00800 31293607 PMC6603150

[B41] Hulsen T. De Vlieg J. Alkema W. (2008). BioVenn – a web application for the comparison and visualization of biological lists using area-proportional Venn diagrams. *BMC Genomics* 9:488. 10.1186/1471-2164-9-488 18925949 PMC2584113

[B42] Inglis P. W. Ciampi A. Y. Salomão A. N. Costa T. D. S. A. Azevedo V. C. R. (2014). Expression of stress-related genes in zebrawood (Astronium fraxinifolium, Anacardiaceae) seedlings following germination in microgravity. *Genet. Mol. Biol.* 37 81–92. 24688295 10.1590/s1415-47572014000100014 PMC3958331

[B43] Jarosz M. Van Lijsebettens M. Woloszynska M. (2020). Plant Elongator-Protein Complex of Diverse Activities Regulates Growth, Development, and Immune Responses. *Int. J. Mol. Sci.* 21:6912. 10.3390/ijms21186912 32971769 PMC7555253

[B44] Jia Y. Tian H. Li H. Yu Q. Wang L. Friml J. (2015). The Arabidopsis thaliana elongator complex subunit 2 epigenetically affects root development. *J. Exp. Bot.* 66 4631–4642. 10.1093/jxb/erv230 25998905 PMC4507768

[B45] Kankel M. W. Ramsey D. E. Stokes T. L. Flowers S. K. Haag J. R. Jeddeloh J. A. (2003). Arabidopsis MET1 cytosine methyltransferase mutants. *Genetics* 163 1109–1122. 12663548 10.1093/genetics/163.3.1109 PMC1462485

[B46] Kanno T. Bucher E. Daxinger L. Huettel B. Böhmdorfer G. Gregor W. (2008). A structural-maintenance-of-chromosomes hinge domain–containing protein is required for RNA-directed DNA methylation. *Nat. Genet.* 40 670–675. 10.1038/ng.119 18425128

[B47] Kenchanmane Raju S. K. Shao M. R. Wamboldt Y. Mackenzie S. (2018). Epigenomic plasticity of Arabidopsis msh1 mutants under prolonged cold stress. *Plant Direct* 2:e00079. 10.1002/pld3.79PLD379 31245744 PMC6508824

[B48] Kolaj-Robin O. Seraphin B. (2017). Structures and Activities of the Elongator Complex and Its Cofactors. *Enzymes* 41 117–149. 10.1016/bs.enz.2017.03.001 28601220

[B49] Korotko U. Chwialkowska K. Sanko-Sawczenko I. Kwasniewski M. (2021). DNA Demethylation in Response to Heat Stress in Arabidopsis thaliana. *Int. J. Mol. Sci.* 22:1555. 10.3390/ijms22041555 33557095 PMC7913789

[B50] Kruse C. P. S. Meyers A. D. Basu P. Hutchinson S. Luesse D. R. Wyatt S. E. (2020). Spaceflight induces novel regulatory responses in Arabidopsis seedling as revealed by combined proteomic and transcriptomic analyses. *BMC Plant Biol.* 20:237. 10.1186/s12870-020-02392-6 32460700 PMC7251690

[B51] Kwon T. Sparks J. A. Nakashima J. Allen S. N. Tang Y. Blancaflor E. B. (2015). Transcriptional response of Arabidopsis seedlings during spaceflight reveals peroxidase and cell wall remodeling genes associated with root hair development. *Am. J. Bot.* 102 21–35. 10.3732/ajb.1400458 25587145

[B52] Laanen P. Saenen E. Mysara M. Van De Walle J. Van Hees M. Nauts R. (2021). Changes in DNA Methylation in Arabidopsis thaliana Plants Exposed Over Multiple Generations to Gamma Radiation. *Front. Plant Sci.* 12:611783. 10.3389/fpls.2021.611783 33868326 PMC8044457

[B53] Labra M. Ghiani A. Citterio S. Sgorbati S. Sala F. Vannini C. (2002). Analysis of Cytosine Methylation Pattern in Response to Water Deficit in Pea Root Tips. *Plant Biol.* 4 694–699. 10.1055/s-2002-37398

[B54] LeFrois C. E. Zhou M. Amador D. M. Sng N. Paul A.-L. Ferl R. (2016). Enabling the Spaceflight Methylome: DNA Isolated from Plant Tissues Preserved in RNAlater Is Suitable for Bisulfite PCR Assay of Genome Methylation. *Gravit. Space Res.* 4 28–37.

[B55] Li B. Dewey C. N. (2011). RSEM: accurate transcript quantification from RNA-Seq data with or without a reference genome. *BMC Bioinformatics* 12:323. 10.1186/1471-2105-12-323 21816040 PMC3163565

[B56] Lopez-Ruiz B. A. Zluhan-Martinez E. Sanchez M. P. Alvarez-Buylla E. R. Garay-Arroyo A. (2020). Interplay between Hormones and Several Abiotic Stress Conditions on Arabidopsis thaliana Primary Root Development. *Cells* 9:2576. 10.3390/cells9122576 33271980 PMC7759812

[B57] Manian V. Orozco-Sandoval J. Diaz-Martinez V. (2021b). Detection of Genes in Arabidopsis thaliana L. Responding to DNA Damage from Radiation and Other Stressors in Spaceflight. *Genes* 12:938. 10.3390/genes12060938 34205326 PMC8234954

[B58] Manian V. Orozco J. Gangapuram H. Janwa H. Agrinsoni C. (2021a). Network Analysis of Gene Transcriptions of Arabidopsis thaliana in Spaceflight Microgravity. *Genes* 12:337. 10.3390/genes12030337 33668919 PMC7996555

[B59] Matzke M. A. Kanno T. Matzke A. J. (2015). RNA-Directed DNA Methylation: the Evolution of a Complex Epigenetic Pathway in Flowering Plants. *Annu. Rev. Plant Biol.* 66 243–267. 10.1146/annurev-arplant-043014-114633 25494460

[B60] Mirouze M. Paszkowski J. (2011). Epigenetic contribution to stress adaptation in plants. *Curr. Opin. Plant Biol.* 14 267–274. 10.1016/j.pbi.2011.03.004 21450514

[B61] Nakashima J. Liao F. Sparks J. A. Tang Y. Blancaflor E. B. (2014). The actin cytoskeleton is a suppressor of the endogenous skewing behaviour of Arabidopsis primary roots in microgravity. *Plant Biol.* 16 142–150. 10.1111/plb.12062 23952736

[B62] Naydenov M. Baev V. Apostolova E. Gospodinova N. Sablok G. Gozmanova M. (2015). High-temperature effect on genes engaged in DNA methylation and affected by DNA methylation in Arabidopsis. *Plant Physiol. Biochem.* 87 102–108. 10.1016/j.plaphy.2014.12.022 25576840

[B63] Nelissen H. Fleury D. Bruno L. Robles P. De Veylder L. Traas J. (2005). The elongata mutants identify a functional Elongator complex in plants with a role in cell proliferation during organ growth. *Proc. Natl. Acad. Sci. U. S. A.* 102:7754. 10.1073/pnas.0502600102 15894610 PMC1140448

[B64] Nelissen H. Groeve S. D. Fleury D. Neyt P. Bruno L. Bitonti M. B. (2010). Plant Elongator regulates auxin-related genes during RNA polymerase II transcription elongation. *Proc. Natl. Acad. Sci.* 107 1678–1683. 10.1073/pnas.0913559107 20080602 PMC2824411

[B65] Niederhuth C. E. Bewick A. J. Ji L. Alabady M. S. Kim K. D. Li Q. (2016). Widespread natural variation of DNA methylation within angiosperms. *Genome Biol.* 17:194. 10.1186/s13059-016-1059-0 27671052 PMC5037628

[B66] Paul A.-L. Amalfitano C. E. Ferl R. J. (2012). Plant growth strategies are remodeled by spaceflight. *BMC Plant Biol.* 12:232. 10.1186/1471-2229-12-232 23217113 PMC3556330

[B67] Paul A.-L. Sng N. J. Zupanska A. K. Krishnamurthy A. Schultz E. R. Ferl R. J. (2017). Genetic dissection of the Arabidopsis spaceflight transcriptome: are some responses dispensable for the physiological adaptation of plants to spaceflight? *PLoS One* 12:e0180186. 10.1371/journal.pone.0180186 28662188 PMC5491145

[B68] Paul A.-L. Zupanska A. K. Schultz E. R. Ferl R. J. (2013). Organ-specific remodeling of the Arabidopsis transcriptome in response to spaceflight. *BMC Plant Biol.* 13:112. 10.1186/1471-2229-13-112 23919896 PMC3750915

[B69] Pikaard S. C. (2013). Methylating the DNA of the Most Repressed: special Access Required. *Mol. Cell* 49 1021–1022. 10.1016/j.molcel.2013.03.013 23541038 PMC3641553

[B70] Raudvere U. Kolberg L. Kuzmin I. Arak T. Adler P. Peterson H. (2019). g:Profiler: a web server for functional enrichment analysis and conversions of gene lists (2019 update). *Nucleic Acids Res.* 47 W191–W198. 10.1093/nar/gkz369 31066453 PMC6602461

[B71] Rigal M. Becker C. Pélissier T. Pogorelcnik R. Devos J. Ikeda Y. (2016). Epigenome confrontation triggers immediate reprogramming of DNA methylation and transposon silencing in Arabidopsis thaliana F1 epihybrids. *Proc. Natl. Acad. Sci. U. S. A.* 113 E2083–E2092. 10.1073/pnas.1600672113 27001853 PMC4833259

[B72] Robinson M. D. Mccarthy D. J. Smyth G. K. (2010). edgeR: a Bioconductor package for differential expression analysis of digital gene expression data. *Bioinformatics* 26 139–140. 10.1093/bioinformatics/btp616 19910308 PMC2796818

[B73] Salmi M. L. Roux S. J. (2008). Gene expression changes induced by space flight in single-cells of the fern Ceratopteris richardii. *Planta* 229 151–159. 10.1007/s00425-008-0817-y 18807069

[B74] Saze H. Scheid O. M. Paszkowski J. (2003). Maintenance of CpG methylation is essential for epigenetic inheritance during plant gametogenesis. *Nat. Genet.* 34:65. 10.1038/ng1138 12669067

[B75] Schüler O. Hemmersbach R. Böhmer M. (2015). A Bird’s-Eye View of Molecular Changes in Plant Gravitropism Using Omics Techniques. *Front. Plant Sci.* 6:1176. 10.3389/fpls.2015.01176 26734055 PMC4689802

[B76] Sewelam N. Oshima Y. Mitsuda N. Ohme-Takagi M. (2014). A step towards understanding plant responses to multiple environmental stresses: a genome-wide study. *Plant Cell Environ.* 37 2024–2035. 10.1111/pce.12274 24417440

[B77] Silva K. J. P. Brunings A. M. Pereira J. A. Peres N. A. Folta K. M. Mou Z. (2017). The Arabidopsis ELP3/ELO3 and ELP4/ELO1 genes enhance disease resistance in Fragaria vesca L. *BMC Plant Biol.* 17:230. 10.1186/s12870-017-1173-5 29191170 PMC5709926

[B78] Sng N. Callaham J. Ferl R. J. Paul A.-L. (2014). Arabidopsis Thaliana for Spaceflight Applications – Preparing Dormant Biology for Passive Stowage and On Orbit Activation. *Gravit. Space Res.* 2 81–99.

[B79] Stassen J. H. M. Lopez A. Jain R. Pascual-Pardo D. Luna E. Smith L. M. (2018). The relationship between transgenerational acquired resistance and global DNA methylation in Arabidopsis. *Sci. Rep.* 8:14761. 10.1038/s41598-018-32448-5 30283021 PMC6170496

[B80] Stroud H. Greenberg M. V. Feng S. Bernatavichute Y. V. Jacobsen S. E. (2013). Comprehensive Analysis of Silencing Mutants Reveals Complex Regulation of the Arabidopsis Methylome. *Cell* 152 352–364. 10.1016/j.cell.2012.10.054 23313553 PMC3597350

[B81] Sugimoto M. Oono Y. Gusev O. Matsumoto T. Yazawa T. Levinskikh M. A. (2014). Genome-wide expression analysis of reactive oxygen species gene network in Mizuna plants grown in long-term spaceflight. *BMC Plant Biol.* 14:4. 10.1186/1471-2229-14-4 24393219 PMC3927260

[B82] Sun D. Xi Y. Rodriguez B. Park H. J. Tong P. Meong M. (2014). MOABS: model based analysis of bisulfite sequencing data. *Genome Biol.* 15:R38. 10.1186/gb-2014-15-2-r38 24565500 PMC4054608

[B83] Supek F. Bošnjak M. Škunca N. Šmuc T. (2011). REVIGO Summarizes and Visualizes Long Lists of Gene Ontology Terms. *PLoS One* 6:e21800. 10.1371/journal.pone.0021800 21789182 PMC3138752

[B84] Tameshige T. Hirakawa Y. Torii K. U. Uchida N. (2015). Cell walls as a stage for intercellular communication regulating shoot meristem development. *Front. Plant Sci.* 6:324. 10.3389/fpls.2015.00324 26029226 PMC4426712

[B85] Tricker P. J. López C. M. R. Hadley P. Wagstaff C. Wilkinson M. J. (2013). Pre-conditioning the epigenetic response to high vapor pressure deficit increases the drought tolerance of Arabidopsis thaliana. *Plant Signal. Behav.* 8:e25974. 10.4161/psb.25974 24270688 PMC4091208

[B86] Vandenbrink J. P. Herranz R. Medina F. J. Edelmann R. E. Kiss J. Z. (2016). A novel blue-light phototropic response is revealed in roots of Arabidopsis thaliana in microgravity. *Planta* 244 1201–1215. 10.1007/s00425-016-2581-8 27507239 PMC5748516

[B87] Vandenbrink J. P. Herranz R. Poehlman W. L. Alex Feltus F. Villacampa A. Ciska M. (2019). RNA-seq analyses of Arabidopsis thaliana seedlings after exposure to blue-light phototropic stimuli in microgravity. *Am. J. Bot.* 106 1466–1476. 10.1002/ajb2.1384 31709515

[B88] Villagomez-Aranda A. L. Garcia-Ortega L. F. Torres-Pacheco I. Guevara-Gonzalez R. G. (2021). Whole-Genome DNA Methylation Analysis in Hydrogen Peroxide Overproducing Transgenic Tobacco Resistant to Biotic and Abiotic Stresses. *Plants* 10:178. 10.3390/plants10010178 33477999 PMC7835756

[B89] Wang Y. An C. Zhang X. Yao J. Zhang Y. Sun Y. (2013). The Arabidopsis elongator complex subunit2 epigenetically regulates plant immune responses. *Plant Cell* 25 762–776. 10.1105/tpc.113.109116 23435660 PMC3608791

[B90] Woloszynska M. Le Gall S. Van Lijsebettens M. (2016). Plant Elongator-mediated transcriptional control in a chromatin and epigenetic context. *Biochim. Biophys. Acta* 1859 1025–1033. 10.1016/j.bbagrm.2016.06.008 27354117

[B91] Xi Y. Li W. (2009). BSMAP: whole genome bisulfite sequence MAPping program. *BMC Bioinformatics* 10:232. 10.1186/1471-2105-10-232 19635165 PMC2724425

[B92] Xie M. Yu B. (2015). siRNA-directed DNA Methylation in Plants. *Curr. Genomics* 16 23–31. 10.2174/1389202915666141128002211 25937811 PMC4412961

[B93] Xu P. Chen H. Jin J. Cai W. (2018). Single-base resolution methylome analysis shows epigenetic changes in Arabidopsis seedlings exposed to microgravity spaceflight conditions on board the SJ-10 recoverable satellite. *NPJ Micr.* 4:12. 10.1038/s41526-018-0046-z 30038957 PMC6043569

[B94] Yong-Villalobos L. Cervantes-Perez S. A. Gutierrez-Alanis D. Gonzales-Morales S. Martinez O. Herrera-Estrella L. (2016). Phosphate starvation induces DNA methylation in the vicinity of cis-acting elements known to regulate the expression of phosphate-responsive genes. *Plant Signal. Behav.* 11:e1173300. 10.1080/15592324.2016.1173300 27185363 PMC4977460

[B95] Yong-Villalobos L. González-Morales S. I. Wrobel K. Gutiérrez-Alanis D. Cervantes-Peréz S. A. Hayano-Kanashiro C. (2015). Methylome analysis reveals an important role for epigenetic changes in the regulation of the Arabidopsis response to phosphate starvation. *Proc. Natl. Acad. Sci. U. S. A.* 112 E7293–E7302. 10.1073/pnas.1522301112 26668375 PMC4702951

[B96] Yu A. Lepère G. Jay F. Wang J. Bapaume L. Wang Y. (2013). Dynamics and biological relevance of DNA demethylation in Arabidopsis antibacterial defense. *Proc. Natl. Acad. Sci. U. S. A.* 110 2389–2394. 10.1073/pnas.1211757110 23335630 PMC3568381

[B97] Zhang X. (2012). Dynamic differential methylation facilitates pathogen stress response in Arabidopsis. *Proc. Natl. Acad. Sci. U. S. A.* 109 12842–12843. 10.1073/pnas.1210292109 22837399 PMC3420173

[B98] Zhang Y. Harris C. J. Liu Q. Liu W. Ausin I. Long Y. (2018). Large-scale comparative epigenomics reveals hierarchical regulation of non-CG methylation in Arabidopsis. *Proc. Natl. Acad. Sci. U. S. A.* 115 E1069–E1074. 10.1073/pnas.1716300115 29339507 PMC5798360

[B99] Zhang Y. Wang L. Xie J. Zheng H. (2015). Differential protein expression profiling of Arabidopsis thaliana callus under microgravity on board the Chinese SZ-8 spacecraft. *Planta* 241 475–488. 10.1007/s00425-014-2196-x 25374148

[B100] Zhong X. Du J. Hale C. J. Gallego-Bartolome J. Feng S. Vashisht A. A. (2014). Molecular mechanism of action of plant DRM de novo DNA methyltransferases. *Cell* 157 1050–1060. 10.1016/j.cell.2014.03.056 24855943 PMC4123750

[B101] Zhou M. Sng N. J. Lefrois C. E. Paul A.-L. Ferl R. J. (2019). Epigenomics in an extraterrestrial environment: organ-specific alteration of DNA methylation and gene expression elicited by spaceflight in Arabidopsis thaliana. *BMC Genomics* 20:205. 10.1186/s12864-019-5554-z 30866818 PMC6416986

[B102] Zhou X. Hua D. Chen Z. Zhou Z. Gong Z. (2009). Elongator mediates ABA responses, oxidative stress resistance and anthocyanin biosynthesis in Arabidopsis. *Plant J.* 60 79–90. 10.1111/j.1365-313X.2009.03931.x 19500300

[B103] Zupanska A. K. Denison F. C. Ferl R. J. Paul A.-L. (2013). Spaceflight engages heat shock protein and other molecular chaperone genes in tissue culture cells of *Arabidopsis thaliana*. *Am. J. Bot.* 100 235–248. 10.3732/ajb.1200343 23258370

