# Supplementary material for: Epigenomic Regulators Elongator Complex Subunit 2 and Methyltransferase 1 Differentially Condition the Spaceflight Response in Arabidopsis
Source: Front Plant Sci. 2021 Sep 13;12:691790. doi: 10.3389/fpls.2021.691790 (PMC8475764; doi:10.3389/fpls.2021.691790)
Supplement: Supplementary file 1 [file Data_Sheet_1.zip › Supplementary File S3.PDF]

File S3 – Harvest images for 11d plates arranged by genotype and environment

Ground Control *elp2-5*

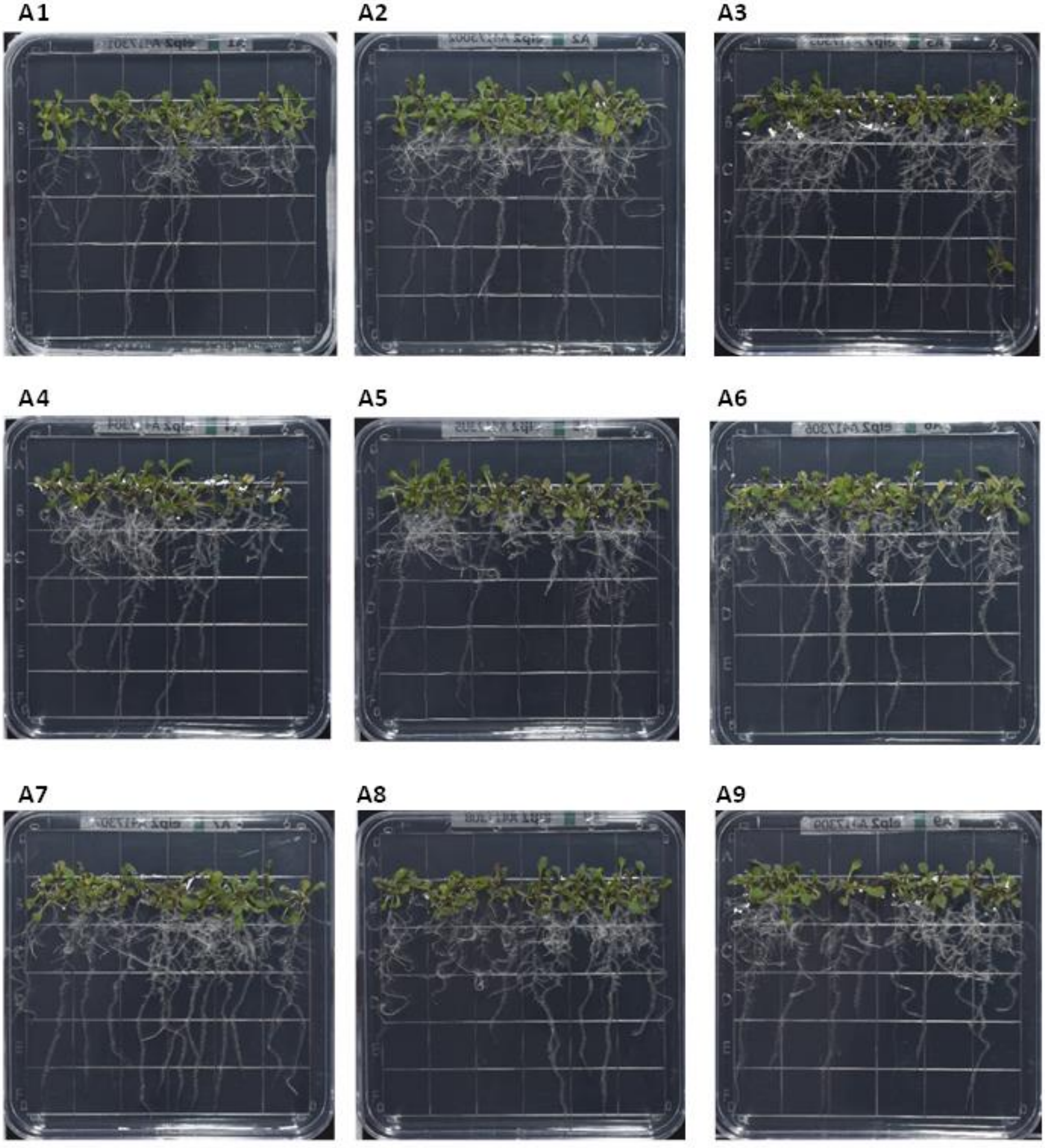

Ground Control *elp2-5*

File S3 – Harvest images for 11d plates arranged by genotype and environment

Spaceflight *elp2-5*

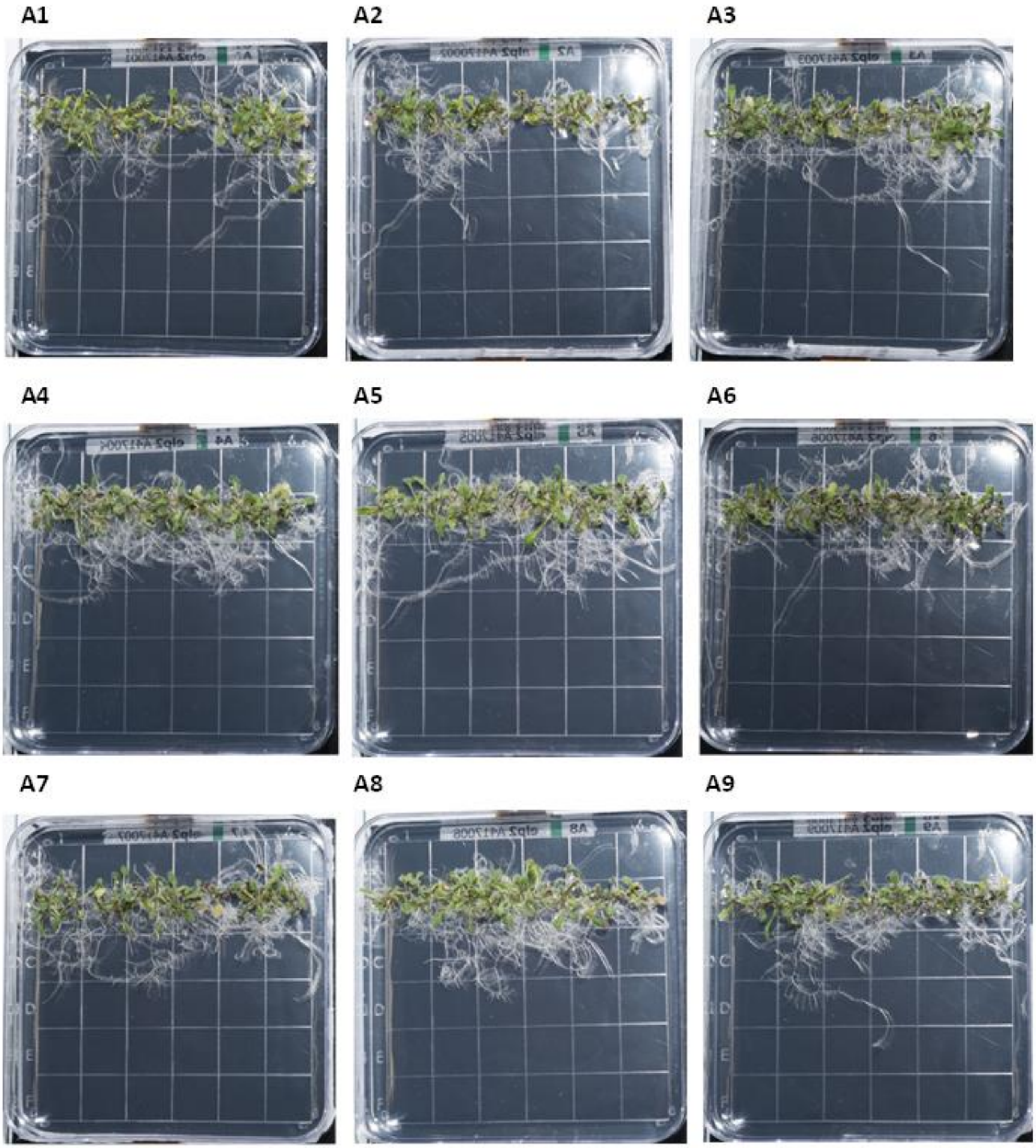

Spaceflight *elp2-5*

File S3 – Harvest images for 11d plates arranged by genotype and environment
Ground Control Col-0

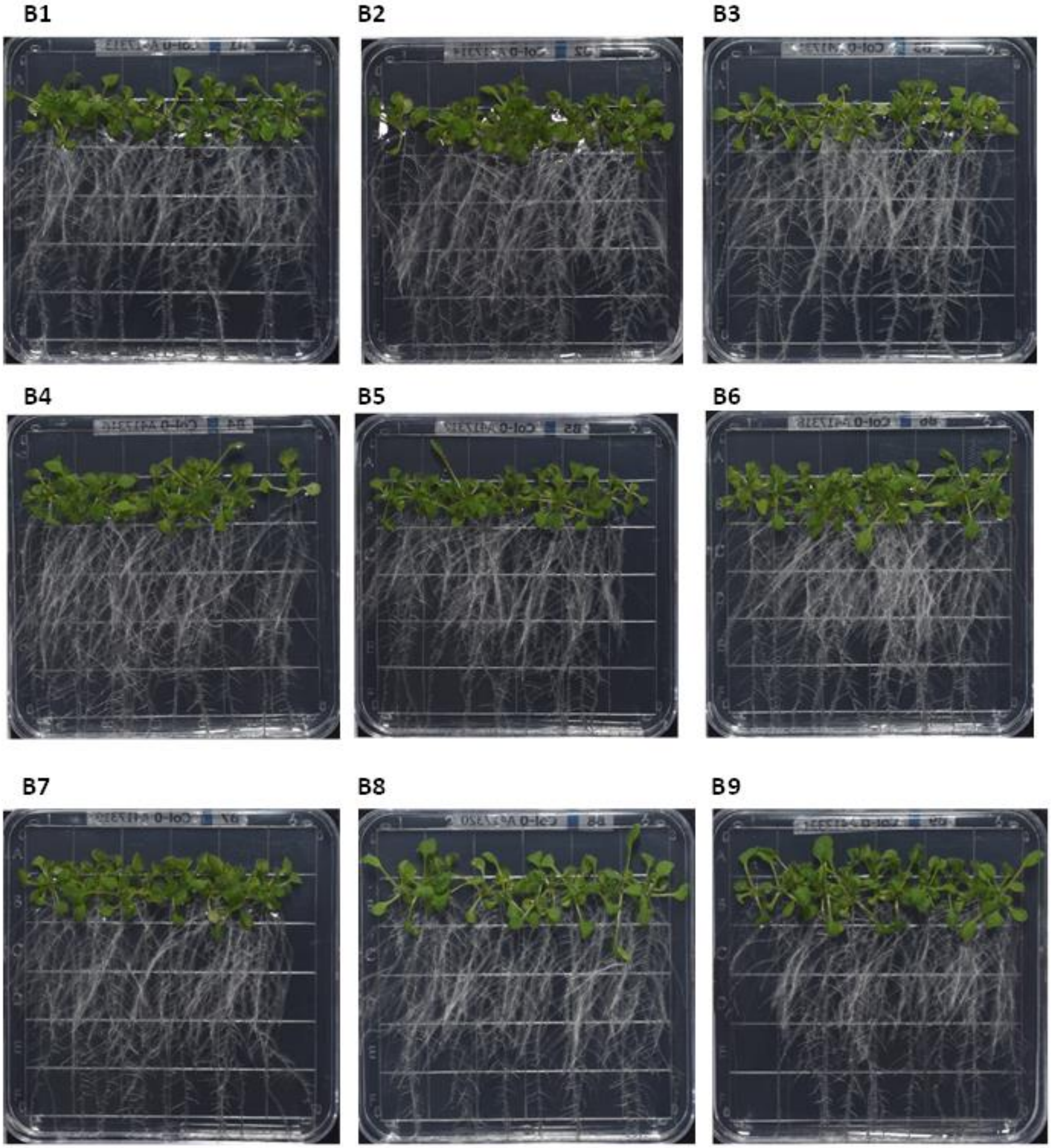

Ground Control Col-0

File S3 – Harvest images for 11d plates arranged by genotype and environment

Spaceflight Col-0

B1

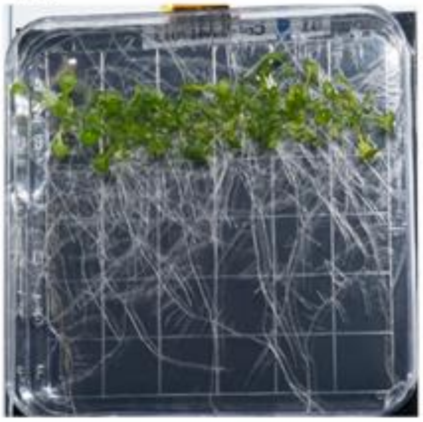

B2

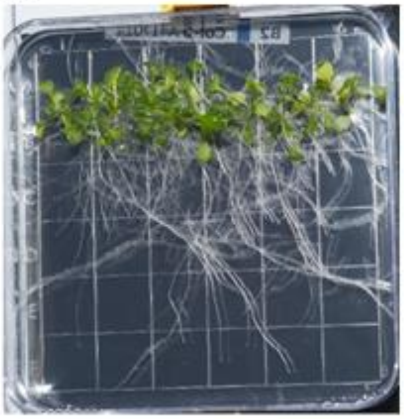

B3

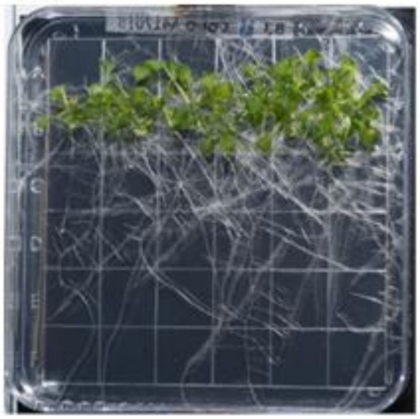

B4

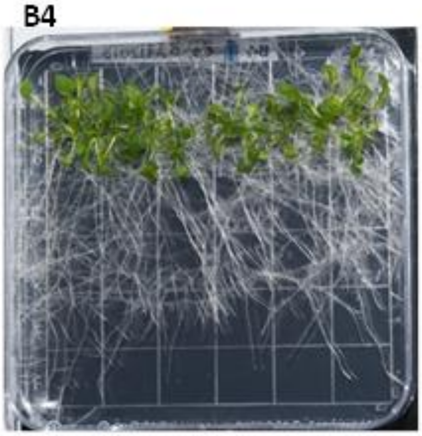

B5

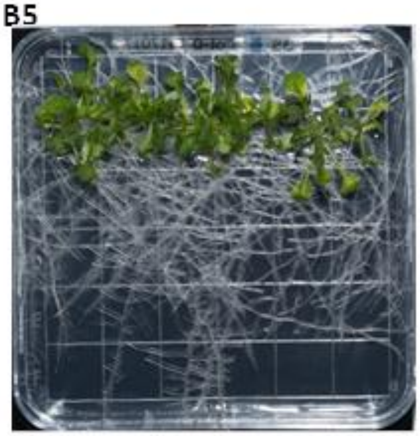

B6

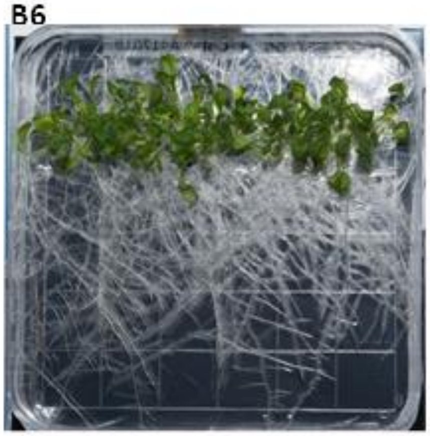

B7

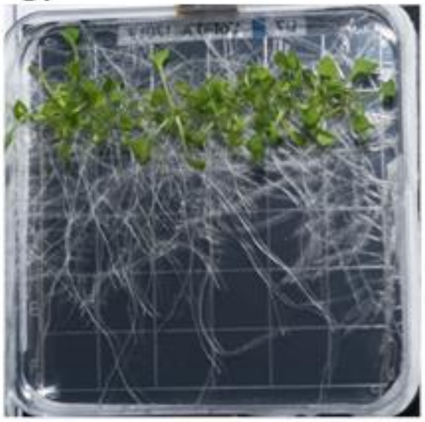

B8

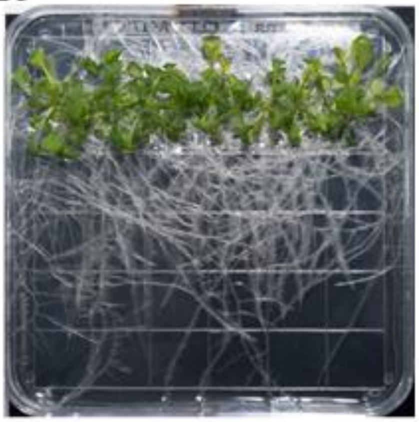

B9

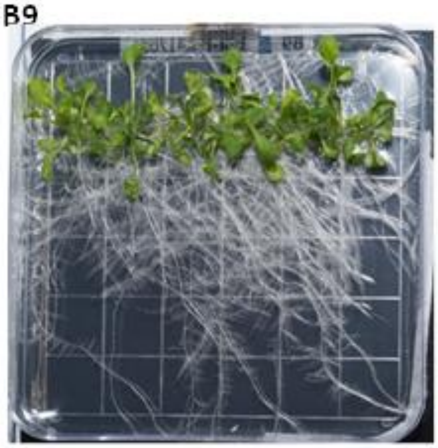

Spaceflight Col-0

File S3 – Harvest images for 11d plates arranged by genotype and environment

Ground Control *met1-7*

C1

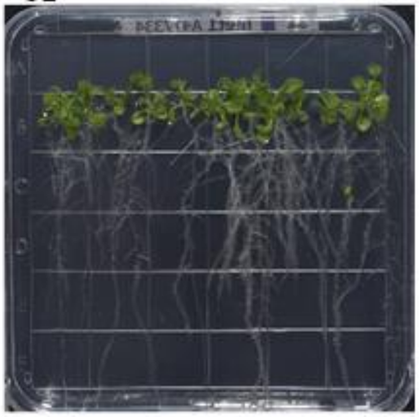

C2

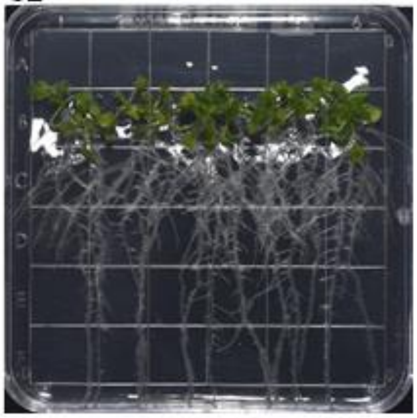

C3

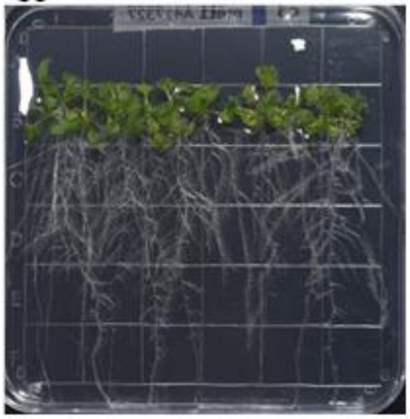

C4

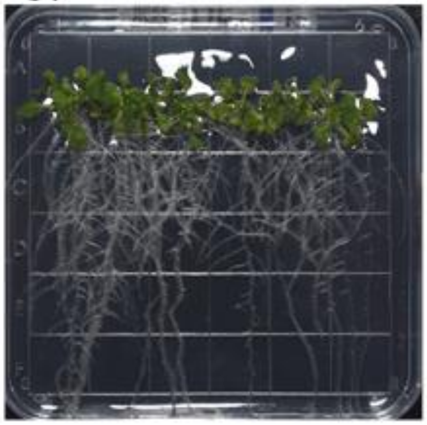

C5

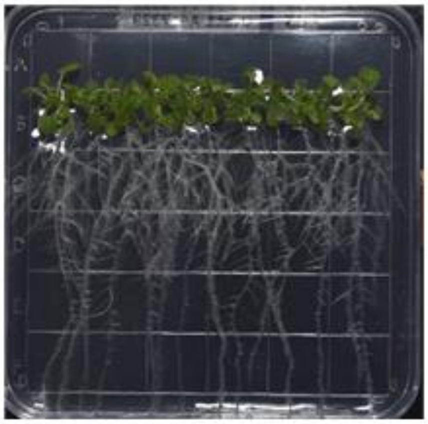

C6

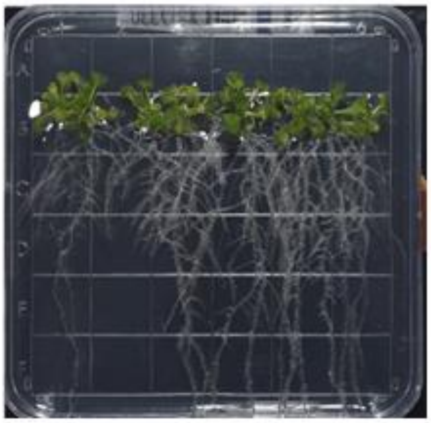

C7

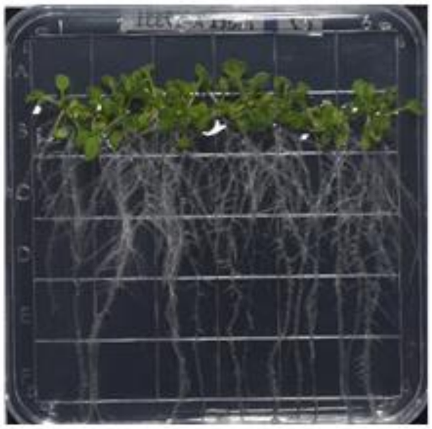

C8

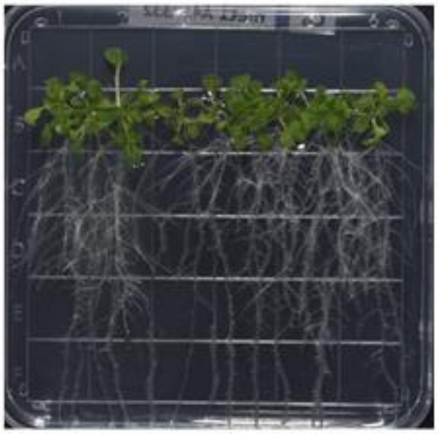

C9

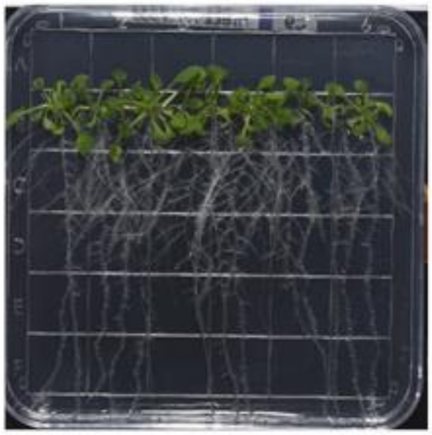

Ground Control *met1-7*

File S3 – Harvest images for 11d plates arranged by genotype and environment

Spaceflight *met1-7*

C1

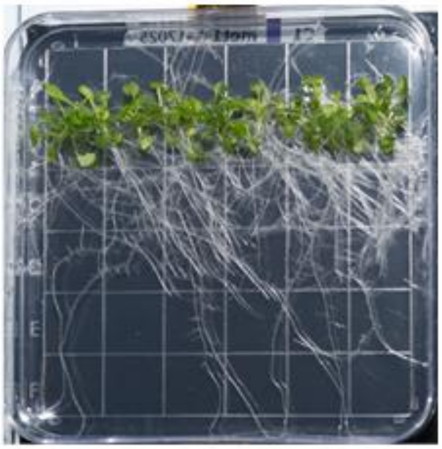

C2

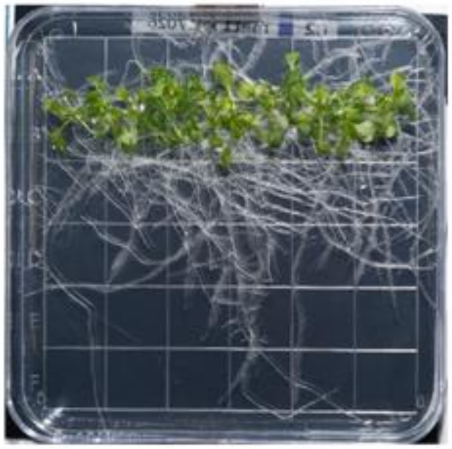

C3

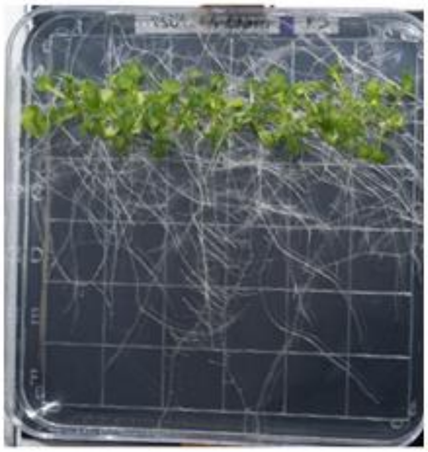

C4

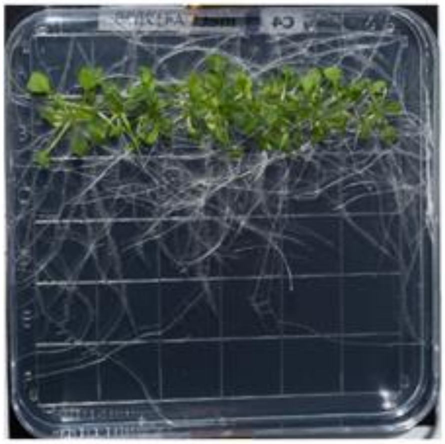

C5

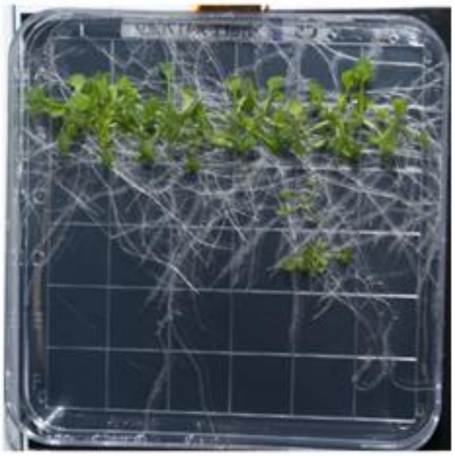

C6

C7

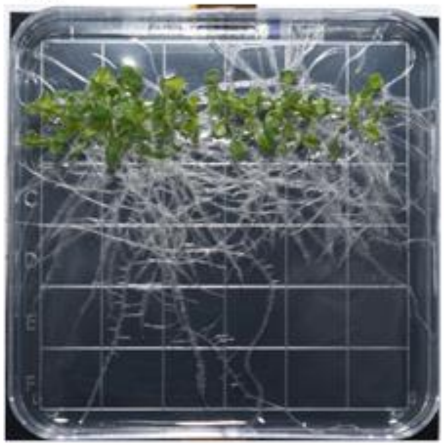

C8

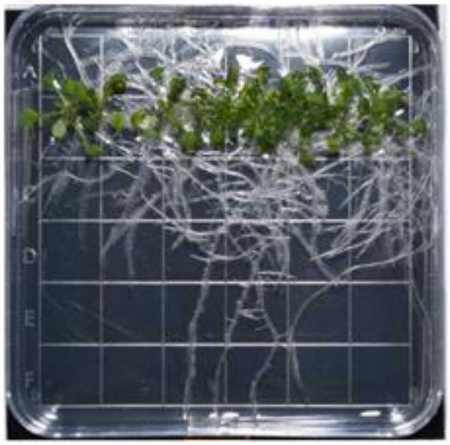

C9

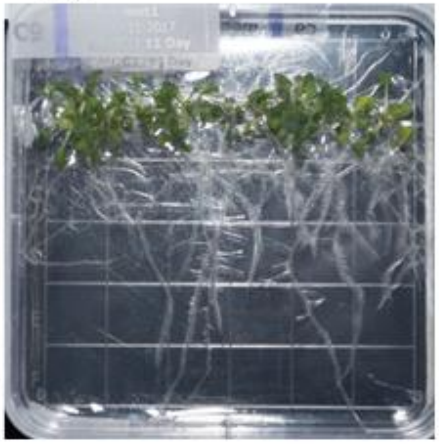

Spaceflight *met1-7*
