# Supplementary material for: Epigenomic Regulators Elongator Complex Subunit 2 and Methyltransferase 1 Differentially Condition the Spaceflight Response in Arabidopsis
Source: Front Plant Sci. 2021 Sep 13;12:691790. doi: 10.3389/fpls.2021.691790 (PMC8475764; doi:10.3389/fpls.2021.691790)
Supplement: Supplementary file 1 [file Data_Sheet_1.zip › Supplementary Table S1.DOCX]

**Table S1.** A comparison of the APEX04 DmC-DEGs with selected gene function databases.

| **Cell Wall Associated Genes** | |
| --- | --- |
| AT4G24000 | CslG2, Cellulose synthase like genes |
| AT3G06260 | GATL4, Glycosyl transferases |
| AT1G11590 | pectin methylesterase, putative |
| AT4G25140 | oleosin 1 |
| AT5G64100 | Peroxidases |
| AT4G30170 | Peroxidases |
| AT5G66870 | ASYMMETRIC LEAVES 2-like 1 |
| AT3G09640 | ascorbate peroxidase 2 |
| AT1G14540 | Peroxidases |
| AT1G14550 | Peroxidases |
| AT1G19250 | flavin-dependent monooxygenase 1 |
| AT1G21120 | COMT-like3 |
| AT1G26250 | hydroxyproline-rich glycoprotein family protein (HRGPs) |
| AT2G26560 | phospholipase A 2A |
| AT3G03670 | Peroxidases |
| AT4G14630 | germin-like protein 9 |
| AT5G39580 | Peroxidases |
| AT1G69730 | Wall-associated kinase family protein; |
| AT3G61810 | glycosyl hydrolase family 17 protein |
| AT3G51350 | Aspartyl Proteases |
| AT4G35770 | Rhodanese/Cell cycle control phosphatase superfamily protein |
| AT5G26300 | Unknown GPI-anchored proteins |
| AT5G41290 | RLK3-like |
| AT2G19900 | NADP-malic enzyme 1 |
|  |  |
| **ROS associate genes** | |
| AT4G14690 | Encodes an early light-induced protein ELIPs. |
| AT5G52640 | Encodes a cytosolic heat shock protein AtHSP90.1 |
| AT1G72660 | P-loop containing nucleoside triphosphate hydrolases superfamily protein. |
| AT4G03320 | Component of the TIC (translocon inner envelope membrane of chloroplasts) |
| AT1G29720 | Leucine-rich repeat transmembrane protein kinase |
| AT5G13930 | TRANSPARENT TESTA 4 (TT4) |
| AT5G42760 | Leucine carboxyl methyltransferase |
| AT2G26150 | Member of Heat Stress Transcription Factor (Hsf) family |
| AT5G51440 | HSP20-like chaperones superfamily protein |
| AT2G47520 | Member of the ERF (ethylene response factor) subfamily B-2 of ERF/AP2 |
| AT3G09640 | Encodes a cytosolic ascorbate peroxidase APX2 |
| AT1G27730 | Related to Cys2/His2-type zinc-finger proteins found in higher plants |
| AT3G48850 | phosphate transporter 3;2 (PHT3;2) |
| AT4G21380 | Putative receptor-like serine/threonine protein kinases |
| AT4G24570 | Encodes one of the mitochondrial dicarboxylate carriers (DIC) |
| AT5G38900 | Thioredoxin superfamily protein; protein disulfide oxidoreductase activity |
| AT5G59220 | highly ABA-induced PP2C gene 1 (HAI1) |
| AT3G08970 | J domain protein localized in ER lumen. |
| AT5G64510 | Tunicamycin Induced 1(TIN1), a plant-specificc ER stress-inducible protein. |
| AT4G35770 | SENESCENCE 1 (SEN1) |
| AT5G07330 | unknown protein |
|  |  |
| **Defense and pathogen associated genes** | |
| AT5G13320 | Auxin-responsive GH3 family protein |
| AT4G12470 | azelaic acid induced 1 |
| AT1G19250 | flavin-dependent monooxygenase 1 |
| AT2G26560 | phospholipase A 2A |
| AT1G75040 | pathogenesis-related gene 5 |
| AT1G75830 | Low-molecular-weight cysteine-rich 67; Predicted PR (pathogenesis-related) protein. |

**Citations for Table S2 Comparisons.**

Tanz, S.K., Castleden, I., Hooper, C.M., Vacher, M., Small, I., and Millar, H.A. (2013). SUBA3: a database for integrating experimentation and prediction to define the SUBcellular location of proteins in Arabidopsis. *Nucleic Acids Res* 41, D1185-1191.

Wang, S., Yin, Y., Ma, Q., Tang, X., Hao, D., and Xu, Y. (2012). Genome-scale identification of cell-wall related genes in Arabidopsis based on co-expression network analysis. *BMC plant biology* 12**,** 138-138.

Willems, P., Mhamdi, A., Simon, S., Storme, V., Kerchev, P.I., Noctor, G., Gevaert, K., and Van Breusegem, F. (2016). The ROS Wheel: refining ROS transcriptional footprints in Arabidopsis. *Plant physiology*.
