# Supplementary material for: Epigenomic Regulators Elongator Complex Subunit 2 and Methyltransferase 1 Differentially Condition the Spaceflight Response in Arabidopsis
Source: Front Plant Sci. 2021 Sep 13;12:691790. doi: 10.3389/fpls.2021.691790 (PMC8475764; doi:10.3389/fpls.2021.691790)
Supplement: Supplementary file 1 [file Data_Sheet_1.zip › Supplementary Table S2.DOCX]

**Table S2.** A comparison of the APEX04 DmC-DEGs with DmC-DEGs of assorted biotic and abiotic stress responses in Arabidopsis.

| **Hewezi et al (2017). Cyst Nematode Parasitism; Root Epigenome** | |
| --- | --- |
| AT3G23510 | Cyclopropane-fatty-acyl-phospholipid synthase |
| AT4G12550 | Auxin-Induced in Root cultures 1 |
| AT5G26270 | unknown protein |
| AT5G33355 | Defensin-like (DEFL) family protein |
| AT5G36150 | putative pentacyclic triterpene synthase 3 |
| AT5G37990 | S-adenosyl-L-methionine-dependent methyltransferases superfamily protein |
| AT5G39110 | RmlC-like cupins superfamily protein |
| AT5G52710 | Copper transport protein family |
|  |  |
| **Korotko et al (2021). DNA Demethylation in Response to Heat Stress** | |
| AT1G48130 | 1-Cys peroxiredoxin PER1 |
| AT1G73190 | Aquaporin TIP3-1 |
| AT2G21490 | Probable dehydrin LEA |
| AT2G34700 | Pollen Ole e 1 allergen and extensin family protein |
| AT3G21720 | Isocitrate lyase |
| AT3G44300 | NIT2 |
| AT4G14690 | Early light-induced protein 2, chloroplastic |
| AT4G25140 | Oleosin |
| AT4G27140 | 2S seed storage protein 1 |
| AT4G27150 | SESA2 |
| AT4G28520 | 12S seed storage protein CRC |
| AT4G34520 | 3-ketoacyl-CoA synthase |
| AT5G07330 | At5g07330 |
| AT5G13930 | Chalcone synthase family protein |
| AT5G52640 | Heat shock protein 90-1 |
| AT5G59220 | Probable protein phosphatase 2C 78 |
|  |  |
| **Stassen et al (2018) transgenerational acquired resistance and global DNA methylation** | |
| AT4G02280 | Sucrose Synthase 3 |
| AT5G25260 | FLOTILIN2 |
|  |  |
| **Yong-Villalobos et al (2016) DNA methylation and regulation of phosphate-responsive genes** | |
| AT1G08090 | NITRATE TRANSPORTER (NRT2.1) |
| AT1G26250 | Proline-rich extensin-like family protein |
| AT1G26380 | FAD-binding Berberine family protein (FOX1) |
| AT1G26390 | FAD-binding Berberine family protein (FOX2) |
| AT1G26410 | FAD-binding Berberine family protein (FOX4) |
| AT1G26420 | FAD-binding Berberine family protein (FOX5) |
| AT1G34047 | Encodes a defensin-like (DEFL) family protein |
| AT1G62760 | Plant invertase/pectin methylesterase inhibitor superfamily protein |
| AT1G71140 | MATE efflux family protein (DTX14) |
| AT1G73220 | ORGANIC CATION/CARNITINE TRANSPORTER1 (OCT1) |
| AT2G05520 | GLYCINE-RICH PROTEIN 3 (GRP-3) |
| AT2G05540 | Glycine-rich protein family |
| AT2G05580 | Glycine-rich protein family |
| AT2G15220 | Plant basic secretory protein (BSP) family protein |
| AT2G15880 | Leucine-rich repeat (LRR) family protein (PEX3) |
| AT2G19900 | NADP-MALIC ENZYME 1 (NADP-ME1) |
| AT2G24720 | GLUTAMATE RECEPTOR 2.2 (GLR2.2) |
| AT2G26150 | HEAT SHOCK TRANSCRIPTION FACTOR A2 (HSFA2) |
| AT2G26560 | PHOSPHOLIPASE A 2A (PLP2) |
| AT3G01345 | Expressed protein |
| AT3G47480 | Calcium-binding EF-hand family protein (CML47) |
| AT3G54580 | Proline-rich extensin-like family protein |
| AT4G01820 | ATP-BINDING CASSETTE B3 (ABCB3) |
| AT4G04490 | CYSTEINE-RICH RLK (RECEPTOR-LIKE PROTEIN KINASE) 36 (CRK36) |
| AT4G04990 | Protein of unknown function (DUF761) |
| AT4G10500 | 2-oxoglutarate (2OG) and Fe(II)-dependent oxygenase superfamily protein (DLO1) |
| AT4G14368 | Regulator of chromosome condensation (RCC1) family protein |
| AT4G22217 | Encodes a defensin-like (DEFL) family protein |
| AT5G07570 | glycine/proline-rich protein |
| AT5G13320 | AVRPPHB SUSCEPTIBLE 3 (PBS3) |
| AT5G25250 | FLOTILLIN 1 (FLOT1) |
| AT5G25260 | SPFH/Band 7/PHB domain-containing membrane-associated protein family FLOT2 |
| AT5G26270 | unknown protein |
| AT5G38910 | RmlC-like cupins superfamily protein |
| AT5G39110 | RmlC-like cupins superfamily protein |
| AT5G39190 | GERMIN-LIKE PROTEIN 2 (GER2) |
| AT5G52710 | Copper transport protein family |
|  |  |
| **Zhou et al (2019) Organ-specific spaceflight methylome in Arabidopsis cultivar WS** | |
| AT1G26380 | FAD-LINKED OXIDOREDUCTASE 1, FOX1 |
| AT2G15220 | Plant basic secretory protein (BSP) family protein |
| AT2G26150 | HEAT SHOCK TRANSCRIPTION FACTOR A2, HSFA2 |
| AT3G02100 | UDP-Glycosyltransferase superfamily protein |
| AT3G44300 | NITRILASE 2 |
| AT4G12520 | lipid-transfer protein/seed storage 2S albumin superfamily protein |
| AT4G12550 | Auxin-Induced in Root cultures 1 |
| AT4G13420 | Potassium transporter 5; High-affinity potassium transporter |
| AT4G14690 | EARLY LIGHT-INDUCIBLE PROTEIN 2, ELIP2 |
| AT4G24570 | DIC2, DICARBOXYLATE CARRIER 2 |
| AT4G35770 | SENESCENCE 1 (STR15) |
| AT5G24210 | Lipase class 3 family protein |
| AT5G38900 | Protein disulfide isomerase |
| AT5G39670 | Calcium-binding EF-hand family protein (CML45) |

**Citations for Table S1 Comparisons.**

Hewezi, T., Lane, T., Piya, S., Rambani, A., Rice, J.H., and Staton, M. (2017). Cyst Nematode Parasitism Induces Dynamic Changes in the Root Epigenome. *Plant Physiology* 174, 405-420.

Korotko, U., Chwialkowska, K., Sanko-Sawczenko, I., and Kwasniewski, M. (2021). DNA Demethylation in Response to Heat Stress in Arabidopsis thaliana. *Int J Mol Sci* 22.

Stassen, J.H.M., Lopez, A., Jain, R., Pascual-Pardo, D., Luna, E., Smith, L.M., and Ton, J. (2018). The relationship between transgenerational acquired resistance and global DNA methylation in Arabidopsis. *Scientific Reports* 8, 14761.

Yong-Villalobos, L., Cervantes-Perez, S.A., Gutierrez-Alanis, D., Gonzales-Morales, S., Martinez, O., and Herrera-Estrella, L. (2016). Phosphate starvation induces DNA methylation in the vicinity of cis-acting elements known to regulate the expression of phosphate-responsive genes. *Plant Signaling & Behavior* 11,

Zhou, M., Sng, N.J., Lefrois, C.E., Paul, A.-L., and Ferl, R.J. (2019). Epigenomics in an extraterrestrial environment: organ-specific alteration of DNA methylation and gene expression elicited by spaceflight in Arabidopsis thaliana. *BMC Genomics* 20, 205.
