# Supplementary figures and images for: Epigenomic Regulators Elongator Complex Subunit 2 and Methyltransferase 1 Differentially Condition the Spaceflight Response in Arabidopsis
Source: Front Plant Sci. 2021 Sep 13;12:691790. doi: 10.3389/fpls.2021.691790 (PMC8475764; doi:10.3389/fpls.2021.691790)

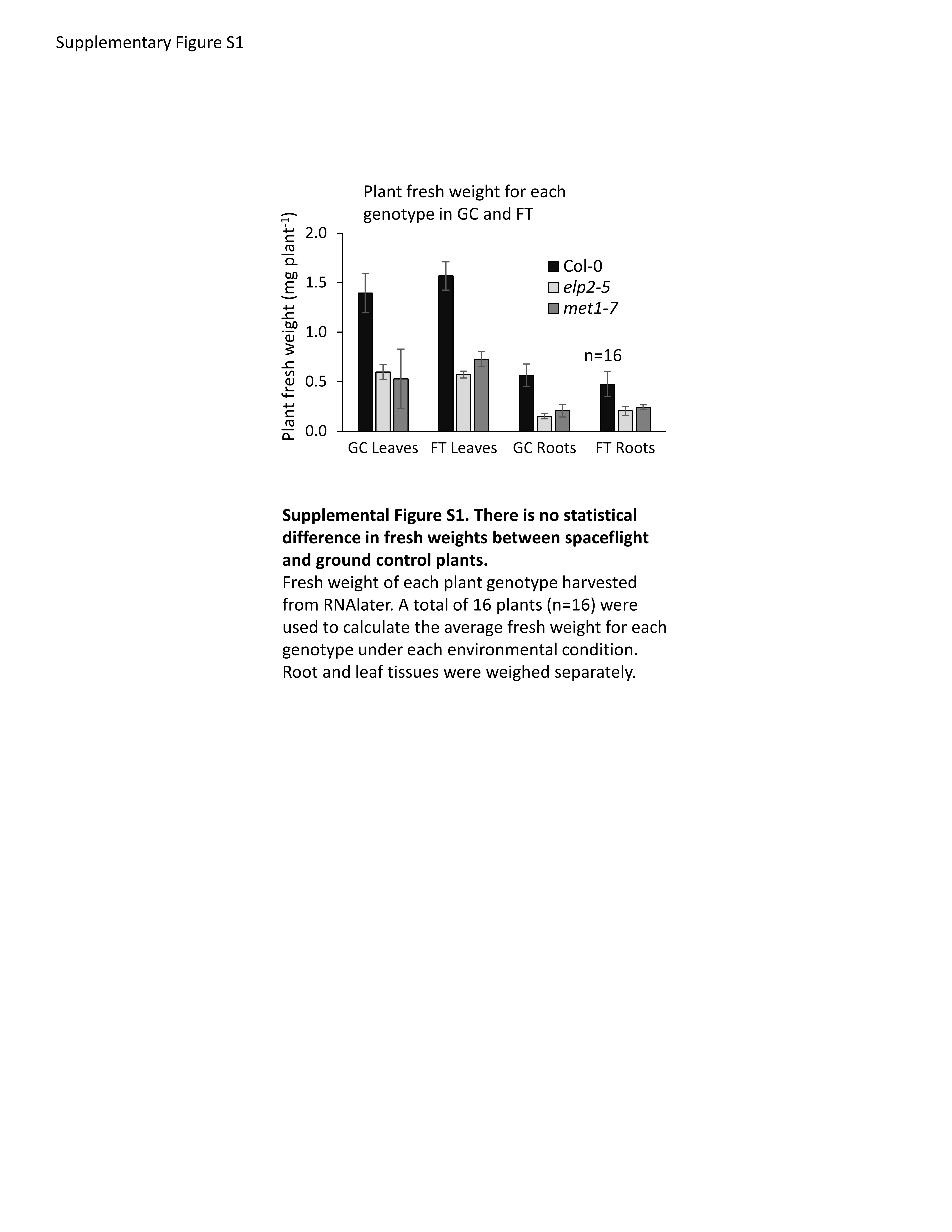

Supplement: Supplementary file 1 [file Data_Sheet_1.zip › Supplementary Figure S1.TIF]

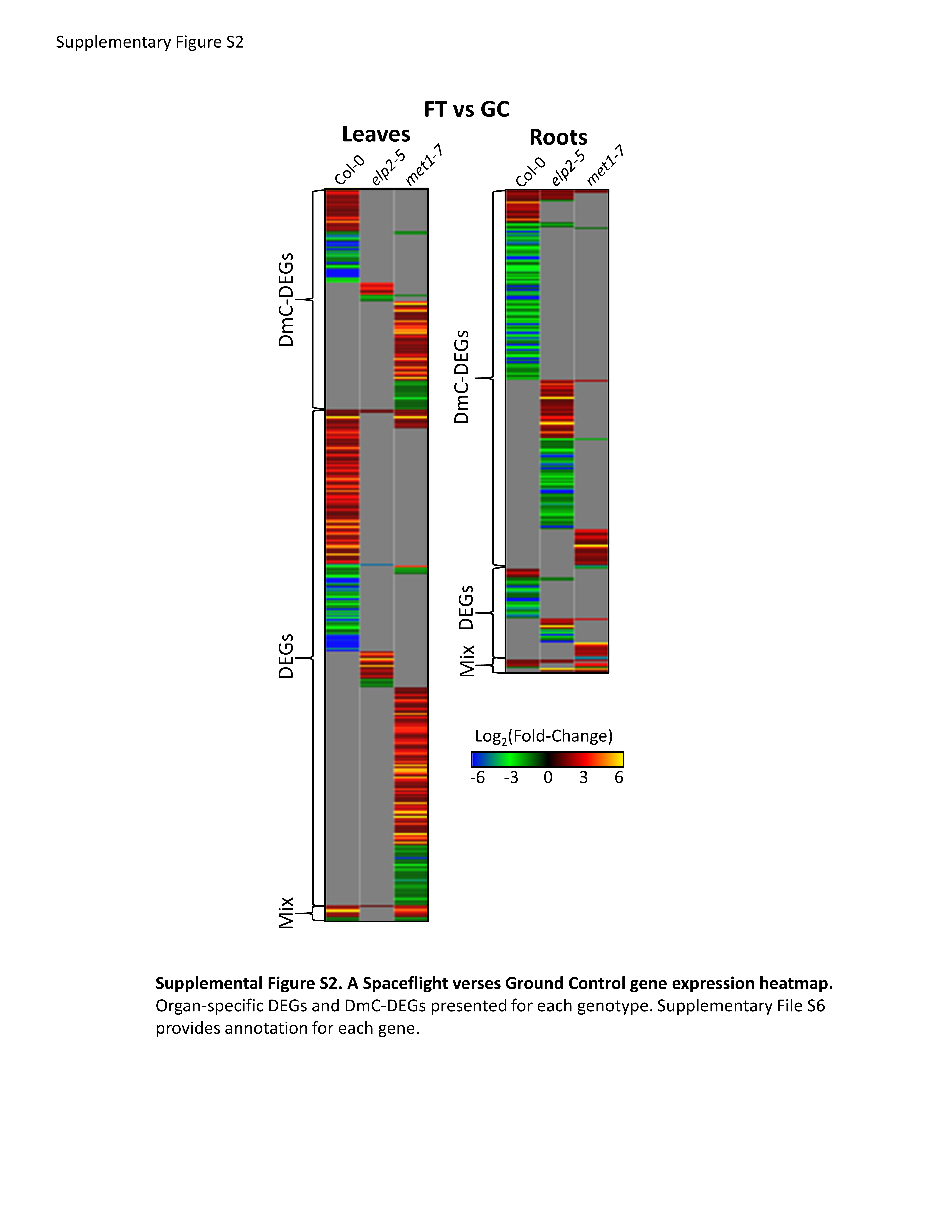

Supplement: Supplementary file 1 [file Data_Sheet_1.zip › Supplementary Figure S2.TIF]

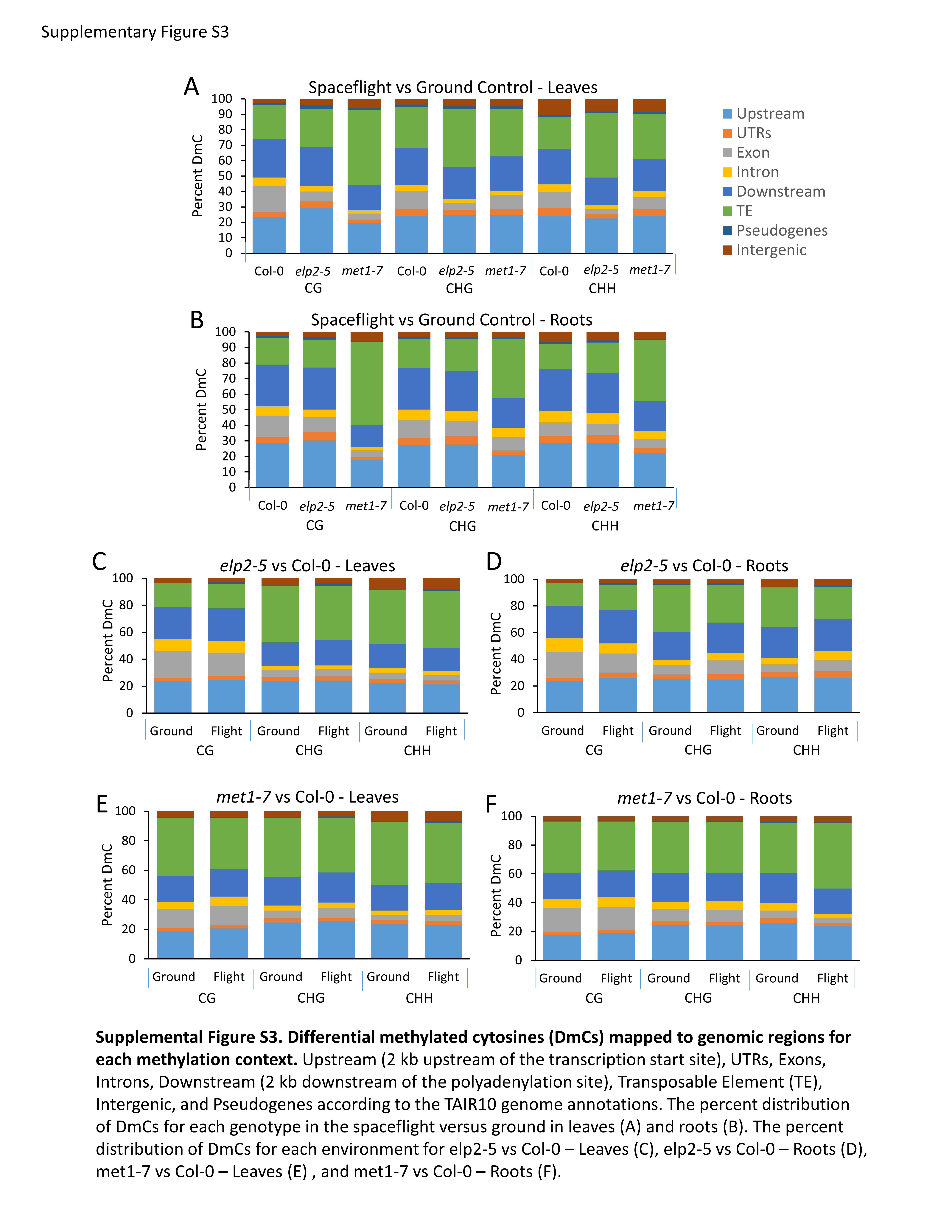

Supplement: Supplementary file 1 [file Data_Sheet_1.zip › Supplementary Figure S3.TIF]
